# Mild hydrothermally treated brewer's spent grain for efficient removal of uranyl and rare earth metal ions†

**DOI:** 10.1039/d0ra08164g

**Published:** 2020-12-22

**Authors:** Yi Su, Wendelin Böhm, Marco Wenzel, Silvia Paasch, Margret Acker, Thomas Doert, Eike Brunner, Thomas Henle, Jan J. Weigand

**Affiliations:** Chair of Inorganic Molecular Chemistry, TU Dresden 01062 Dresden Germany jan.weigand@tu-dresden.de; Chair of Food Chemistry, TU Dresden 01062 Dresden Germany; Chair of Bioanalytical Chemistry, TU Dresden 01062 Dresden Germany; Central Radionuclide Laboratory, TU Dresden 01062 Dresden Germany; Chair of Inorganic Chemistry II, TU Dresden 01062 Dresden Germany

## Abstract

The increasing concerns on uranium and rare earth metal ion pollution in the environment require sustainable strategies to remove them from wastewater. The present study reports an eco-friendly approach to convert a kind of protein-rich biomass, brewer's spent grain (BSG), into effective biosorbents for uranyl and rare earth metal ions. The employed method reduces the energy consumption by performing the hydrothermal treatment at a significantly lower temperature (150 °C) than conventional hydrothermal carbonization. In addition, with the aid of the Maillard reaction between carbohydrates and proteins forming melanoidins, further activation processes are not required. Treatment at 150 °C for 16 h results in an altered biosorbent (ABSG) with increased content of carboxyl groups (1.46 mmol g^−1^) and a maximum adsorption capacity for La^3+^, Eu^3+^, Yb^3+^ (pH = 5.7) and UO_2_^2+^ (pH = 4.7) of 38, 68, 46 and 221 mg g^−1^, respectively. Various characterization methods such as FT-IR, ^13^C CP/MAS NMR, SEM-EDX and STA-GC-MS analysis were performed to characterize the obtained material and to disclose the adsorption mechanisms. Aside from oxygen-containing functional groups, nitrogen-containing functional groups also contribute to the adsorption. These results strongly indicate that mild hydrothermal treatment of BSG could be applied as a greener, low-cost method to produce effective adsorbents for uranyl and rare earth metal ion removal.

## Abstract 2

Effective biosorbent ABSG is obtained *via* hydrothermal treatment of BSG at low temperature without activation, minimizing energy consumption and environmental impact.

## Graphical abstract

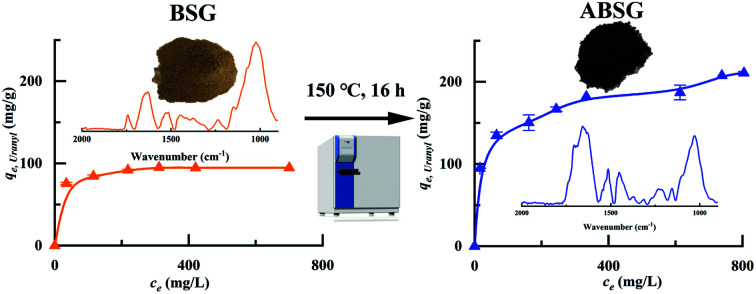

## Introduction

Uranium is one of the most widely used radioactive elements and an unreplaceable raw material for nuclear energy. In recent years, the concentration of uranium in aqueous environments has increased significantly as a result of human activities, such as mining and nuclear industry.^1^ These unwanted releases of uranium cause severe environmental pollution and health risks due to the chemical and radiologic toxicity of uranium.^2^ Owing to this, the World Health Organization (WHO) has recommended that the maximum concentration of uranium in drinking water should not exceed 30 μg L^−1^.^3^ Therefore, the removal of uranium from wastewater is necessary to protect the environment and health. In addition, rare earth elements are often found associated with uranium especially in wastewater from mining and spent nuclear fuel.^4,5^ It is reported that rare earth elements also can be tissue-specific bioaccumulated, thus causing damage to the lungs, liver and brain.^6^ Considering the health and environment hazards of uranyl and rare earth metal ions, it would be efficient and economical to remove both from wastewater simultaneously.

Recently, adsorption process has risen more and more interests in both academic and industry fields to remove uranyl and rare earth metal ions from wastewater. This is because adsorption demonstrates obvious advantages *e.g.* fast kinetics, high adsorption capacity, remarkable stability and the avoidance of organic solvent. Furthermore, adsorption is considered to be inexpensive and easier to use than other methods.^4^ Different adsorbents have been developed and tested for the adsorption of uranyl and rare earth metal ions, such as graphene oxide-based adsorbent,^7,8^ nanomaterials,^9^ cationic resin,^10^ mesoporous silica-based adsorbent^11^ and biosorbent.^12^ On the base of cost-to-performance ratio, biosorbents are most preferable and economically viable among all the adsorbents, as they are convenient, readily available and cheap.^13^ They also present great potential as an eco-friendly alternative to synthetic adsorbents.^14^ Various kinds of biopolymers have been reported as suitable biosorbents for uranium and rare earth metal ions, such as fungi (yeast (*Saccharomyces cerevisiae*) embedded cellulose, 25.9 mg g^−1^ for Eu^3+^),^15^ bacteria (*Pseudomonas aeruginosa*, 208 μmol g^−1^ dry biomass for La^3+^)^16^ and cellulose nanofiber (167 mg g^−1^ for UO_2_^2+^).^17^ However, the adsorption capacity of natural biomass is normally lower than that of synthetic adsorbents (*e.g.* graphene oxide-activated carbon felt composite, 298 mg g^−1^ for UO_2_^2+^),^18^ which requires proper modification methods, either by chemical functionalization or thermal conversion.^12^

Comparing to traditional chemical modification methods for biosorbents, hydrothermal treatment presents some unique benefits as a greener and economical approach. It is highly flexible on the choice of feedstock, and there is no need of pre-drying of the biomass feeds and no toxic chemicals are required.^19^ In this context, hydrothermal carbonization (HTC) is the most commonly used hydrothermal process for biosorbent production, and is typically performed at high temperatures of up to 250 °C during which the biomass is submerged in water and heated under pressure.^20^ The resulting solid product, named hydrochar, possesses abundant surface functional groups^21^ and has a high affinity towards uranium and rare earth metal ions. For example, hydrochar prepared from pine needles^22^ and carbonaceous spheres from glucose with AlCl_3_ catalyst^23^ present adsorption capacity for UO_2_^2+^ of 62.7 mg g^−1^ and 163 mg g^−1^, respectively. However, the application of hydrochar is limited by some drawbacks, like high operation temperature and pressure, low porosity, and low specific surface area. Especially, physical or chemical activation is usually required to obtain satisfying adsorption capacity of hydrochars.^24^

The goal of our studies is to develop an adsorbent for UO_2_^2+^ and trivalent rare earth metal ions removal employing brewer's spent grain (BSG), a large-scale produced by-product of the beer production, as a low-cost and convenient manageable raw material. Literatures have reported the application of BSG as a biosorbent for metal ions, such as Mn^2+^, Zn^2+^, Ni^2+^, Cd^2+^, Cu^2+^ and Pb^2+^.^25–28^ Recently, activated hydrochar produced from BSG is reported for the adsorption of acetaminophen.^29^ Nevertheless, it is worth to notice that the activated hydrochar was obtained at 220 °C for 16 h with a chemical activation process using KOH and high temperature (800 °C). Both, high temperature and the strong alkaline condition inevitably undermine the environmental benefits of the hydrochar.

The common starting materials for HTC processes are lignocellulosic materials, while BSG could be described as a biomass rich in lignocellulose and protein.^30^ It is thus possible that the Maillard reaction takes place at an elevated temperature and the resulting products contribute to enhance the adsorption capacity.^31^ Based on this hypothesis, mild hydrothermal conditions (temperature of 150 °C) are evaluated in the present work to enhance the adsorption capacity of BSG with the aid of Maillard reaction. The results show that Maillard reaction between carbohydrates and proteins with the formation of melanoidins plays an important role in the enhanced presence of functional groups suitable for metal coordination and yields in high adsorption capacity of the altered biosorbent (ABSG) towards UO_2_^2+^ without further treatment. This minimizes energy consumption and makes it possible to dispense additional activation processes, so that current disadvantages of the HTC process are overcome, indicating a simpler and eco-friendly route for the production of biosorbents. Effects of reaction temperature and time on the adsorption capacity, the properties of products and the Maillard reaction were investigated to shed light on the involved reaction mechanisms. Furthermore, spectroscopic and thermogravimetric methods were employed to fully characterize the obtained biosorbents. The adsorption properties towards UO_2_^2+^ and La^3+^, Eu^3+^ and Yb^3+^ as representatives of the early, middle and late lanthanides onto BSG-based biosorbents were examined including the adsorption mechanisms.

## Experimental

### Materials

NaOH (97%, VWR chemicals), La(NO_3_)_3_·6H_2_O (99.9%, Thermo Fisher GmbH), Eu(NO_3_)_3_·5H_2_O (99.9%, Sigma-Aldrich), Yb(NO_3_)_3_·5H_2_O (99.9%, Sigma-Aldrich), UO_2_(CH_3_COO)_2_·2H_2_O (Merck), HNO_3_ (supra pure, 69%, Carl Roth GmbH), Na_2_CO_3_ (99.5%, Grüssing GmbH), NaHCO_3_ (99%, Grüssing GmbH) and potassium hydrogen phthalate (Laborchemie Apolda GmbH) were applied as purchased. Ultra-pure water (18.2 MΩ cm^−1^, arium® pro, Sartorius) was used in all experiments.

### Preparation of standardized brewer's spent grain

Brewer's spent grain (BSG, water content 78 wt%) was obtained from our laboratory-scale brewery plant (Technical University of Dresden, Germany) during the production of a Pilsner beer directly after the mashing process. 14.6 kg *Pilsner malt* (Weyermann) was used in this brewing. During mashing process, 53 L of water was poured initially, with a replenishment volume of 58 L. The temperature and time of different mashing procedures are summarized in Table S1 in the ESI.† The fresh BSG was then stored at −16 °C until further processing. For the preparation of standardized BSG, the material was defrosted at room temperature and dried at 60 °C under reduced pressure (<70 mbar) for 72 h (water content 3.0 wt%). Afterwards the BSG was milled using a coffee grinder (MayOcean) for 30 s, left to rest for 10 s and milled again for 20 s. The milled BSG was passed through a 315 μm sieve, characterized and used for the adsorption studies.

### Hydrothermal treatment of brewer's spent grain (BSG) to yield altered biosorbent (ABSG)

Hydrothermal treatment of BSG was conducted in an autoclave reactor (DAB-3, Berghof Products + Instruments GmbH) with 250 mL volume. Typically, 15 g fresh, defrosted BSG was mixed manually inside the reactor with 10 mL ultra-pure water and the pH was adjusted to 10 using 0.01 M NaOH. The reactor was heated in an oven (Dry-line 115, VWR International) at different temperatures (100 °C, 125 °C, 150 °C, 175 °C) for various time periods (1 h, 4 h, 8 h, 16 h, 24 h). After reaction, the solid product was collected and dried at 60 °C under reduced pressure (<70 mbar) overnight to reduce the water content to less than 5 wt%, crushed and passed through 315 μm sieve to obtain a homogeneous fraction. The hydrothermal treated BSG was designated with ABSG-*θ*, *t*, where *θ* is the reaction temperature and *t* is the reaction time, in order to investigate the influence of the hydrothermal treatment parameters. For general characterization (^13^C solid state NMR, oxygen functional groups and STA-GC-MS analysis) and adsorption study, ABSG (ABSG-150 °C, 16 h) was used with a water content of 4.3 wt%, N content of 5.0 wt% and an estimate protein content of 29.2 wt%.

### Adsorption study

Batch adsorption experiments for BSG and ABSG were performed by suspending 2 mg dry adsorbent in 2 mL solution containing the metal ions of the required concentration in micro centrifuge tubes (2 cm^3^, Safe-Lock, Eppendorf) with an overhead shaker (Reax 2, Heidolph) at room temperature. For experiments using ^169^YbCl_3_ radiotracer, 1 mg adsorbent and 1 mL metal solution was used. The kinetic studies were conducted under constant initial concentration at pH = 5.7 for La^3+^, Eu^3+^ and Yb^3+^ and at pH = 4.7 for UO_2_^2+^ with a series of time intervals ranging from 0 to 180 min. 0.1 M or 1.0 M HNO_3_ was used to adjust the initial pH in the range of 1–6 for La^3+^, Eu^3+^ and Yb^3+^ and 1–5 for UO_2_^2+^. The pH range was carefully chosen in order to prevent precipitation. The equilibrium pH was measured with an InLab micro pH electrode (Mettler Toledo). Adsorption isotherms were obtained with different initial metal concentrations ranging from 100 mg L^−1^ to 1000 mg L^−1^ at pH = 5.7 for La^3+^, Eu^3+^ and Yb^3+^ and at pH = 4.7 for UO_2_^2+^. After adsorption the solution was filtered through a 13 mm syringe filter with a 0.22 μm PTFE film (Fisher Scientific), and the metal concentration before and after adsorption was determined by ICP-OES (OPTIMA 2000DV, PerkinElmer). The adsorption capacity (*q*_e_, mg g^−1^) was calculated using the following equation (eqn (1)):1
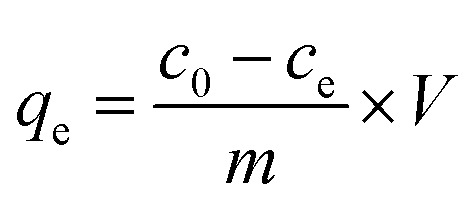
where *c*_0_ (mg L^−1^) and *c*_e_ (mg L^−1^) are the metal concentrations before and after adsorption, *m* (g) is the mass of adsorbent and *V* (L) is the volume of metal solution.

Adsorption experiments using ^169^Yb radiotracer (YbCl_3_, Polatom) were also performed. In these experiments, the metal ions distribution between the solution and adsorbent was determined radiometrically employing radiation from ^169^Yb with a NaI (TI) scintillation counter (Hidex AMG, Hidex GmbH). The count rate (CPM, counts per minute) of 0.5 mL of the supernatant liquid and the remaining 0.5 mL supernatant liquid with the adsorbent were determined. The adsorption capacity (*q*_e_, mg g^−1^) is calculated as eqn (2):2
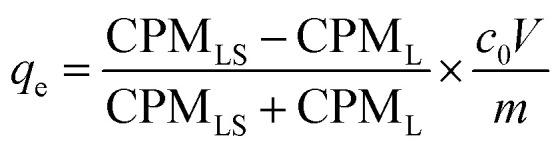
where CPM_LS_, CPM_L_ are the determined count rate for the adsorption sample with and without solid adsorbent, *c*_0_ (mg L^−1^) is the metal concentration before adsorption determined by ICP-OES, *m* (g) is the mass of adsorbent and *V* (L) is the volume of metal solution. All the adsorption experiments were performed in duplicate and the average value and standard deviation are reported.

Effects of temperature on the adsorption capacity of the biosorbents were examined by performing adsorption isotherms at three different temperatures, 25 °C, 45 °C and 65 °C. Generally, 2 mg adsorbent was mixed with 2 mL metal ions solution with different initial concentrations (100–600 mg L^−1^) at pH = 4.7 (UO_2_^2+^) and pH = 5.7 (La^3+^, Eu^3+^ and Yb^3+^) in 10 mL test tube using magnetic stirrer (IKA®, RCT basic) at a stirrer speed of 180 rpm. The temperature was controlled by a circulation thermostat (UH 4, MLW-Medingen). Thermodynamic parameters were calculated according to the following equations (eqn (3) and (4)):

3 Δ*G* = Δ*H*^0^ − *T*Δ*S*^0^

4
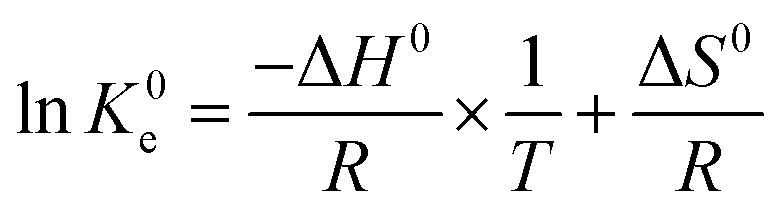
where Δ*G*^0^ (J mol^−1^) is the change in Gibb's energy, Δ*H*^0^ (J mol^−1^) is the change in enthalpy, Δ*S*^0^ (J mol^−1^ K^−1^) is the change in entropy, *T* (K) is the adsorption temperature, *R* (8.3143 J mol^−1^ K^−1^) is the gas constant, and *K*^0^_e_ (/) is the dimensionless thermodynamic equilibrium constant. *K*^0^_e_ is calculated according to literature from the isotherm equilibrium constant (*K*_L_, L mg^−1^) of the best isotherm model fitted, which in current case is Langmuir isotherm model.^32^

### Characterization

BSG and ABSG with a water content of less than 5 wt% were used in all characterizations. Infrared (FT-IR) spectra of adsorbents before and after the metal adsorption were obtained with a single beam Fourier transform infrared VERTEX 70 spectrometer (Bruker). An ATR (attenuated total reflectance) unit (diamond) with a single reflection optics at an interaction angle of 45° was used. The spectra were recorded over the range of 4500 to 400 cm^−1^ with a resolution of 4 cm^−1^, and averaged over 32 scans. In order to investigate the detailed changes of chemical structures, the obtained spectra were processed using the OPUS software package as provided by Bruker to compare the intensity of certain bands. Baseline corrections were applied at 3014 cm^−1^, 2807 cm^−1^, 1783 cm^−1^, 1532 cm^−1^, 1086 cm^−1^, 900 cm^−1^ and 400 cm^−1^. Then the spectra were normalized respecting to –CH_2_– antisymmetric stretching vibration bands (≈2924 cm^−1^). For normalization, the absorbance value of this band was set to 1.0 and the complete spectrum was multiplied by a ratio factor. The raw spectra are provided in Fig. S1 in the ESI† to demonstrate that no obvious intensity changes occurred during the spectra manipulation.

^13^C solid state NMR spectra were recorded on a Bruker Ascend 800 MHz spectrometer using a commercial 3.2 mm MAS NMR probe and operating at a resonance frequency of 201.2 MHz. The MAS frequency was 15 kHz. Adamantane was used as external standard. Ramped ^1^H–^13^C cross-polarization (CP, contact time: 4 ms) and SPINAL ^1^H-decoupling during the signal acquisition was applied. The recycle delay was 3 s. 26 000 scans (BSG)/28 000 scans (ABSG) were accumulated for signal-to-noise improvement.

The specific surface area of BSG and ABSG was determined by the Brunauer–Emmett–Teller (BET) method (SA 9600, Horiba Scientific). The sample was subjected to a pre-treatment at 110 °C for 90 min. The analysis was performed using 8% N_2_ in He. The adsorption took place at −196 °C and the desorption was at room temperature.

Scanning electron microscopy (SEM) and energy-dispersive X-ray spectroscopy (EDX) analysis were performed on a scanning electron microscope (SU8020, HITACHI) equipped with an energy-dispersive X-ray spectrometer X-Max^N^ (OXFORD Instrument) at an electron beam voltage of 20 kV. The metal-loaded ABSG samples were dried at 60 °C under reduced pressure (<70 mbar) for 48 h and coated with a Au layer by a rotary pumped coater (Q150R ES, Quorum) at 5 mA. The surface morphology images of metal loaded ABSG were taken at magnification of 1000 times, and the EDX mapping was taken at 20 kV/10 μA, magnification of 1000–5000 times for 25 frames.

The thermal stability of adsorbents and volatile products produced during decomposition were analyzed by simultaneous thermal analyzer (STA 8000, PerkinElmer) coupled with a GC-MS (GC Clarus 680, MS Clarus SQ 8S, PerkinElmer). The samples were heated from 40 °C to 600 °C with a heating rate of 20 °C min^−1^ under helium atmosphere. The volatile products generated at 375–385 °C (BSG, ABSG) and at 340–360 °C (Yb-ABSG) were detected using GC-MS. The GC temperature was initially set to 35 °C with a hold time of 3 min, and then increased with a heat rate of 5 °C min^−1^ until 220 °C, and hold at 220 °C for 3 min. The obtained MS spectra were interpreted using NIST mass spectral search program provided by PerkinElmer.

The content of Ca, Fe, Mn, Mg, Zn, K, Na, P and Si (mineral elements) of BSG and ABSG were determined by ICP-OES after microwave-assisted digestion. In general, 10 mL HNO_3_ (supra pure, 69%) were added to 0.1 g adsorbent and reacted for 15 min at room temperature before heating for 15 min at 210 °C (MARS 6, CEM GmbH). Elemental analysis were performed on a Vario MICRO cube (Elementar Analysensysteme GmbH) in CHNS mode to determine the content of carbon, nitrogen, hydrogen and sulfur. The oxygen content was calculated by mass balance considering the content of carbon, nitrogen, hydrogen, sulfur and the mineral elements determined by ICP-OES. The results of elemental analysis and mineral elements analysis are provided in Tables S2 and S3 in the ESI.† Protein content was estimated according to nitrogen content by multiplying factor 5.83.^33^

Maillard reaction products (MRPs) were analyzed according to literature with slight modifications.^34^ Typically, the samples were enzymatically hydrolyzed, cleaned up through a solid phase extraction cartridge, and analyzed *via* HPLC-MS/MS (Agilent Technologies). For separation, a HPLC-column (Kinetex-C-18 column, 1.7 μm, 100 Å, 50 × 2.1 mm) was used. Amino acid analysis (ion-exchange chromatography with ninhydrin detection) was performed by the method proposed by Hellwig *et al.*.^35^ Point of zero charge (pH_pzc_) of BSG and ABSG was determined by solid addition method,^36^ using 0.2 g adsorbent suspended in 10 mL 0.1 M NaNO_3_ solution. The initial pH of the latter was adjusted to 1–10 using 0.1 M HNO_3_ or 0.1 M NaOH. The content of oxygen functional groups (OFGs) were quantified using Boehm titration.^37^ In general, a mixture of 0.9 g adsorbents and 50.00 mL of one of the three reaction bases, NaHCO_3_, Na_2_CO_3_ and NaOH, in a concentration of 0.05 M was shaken for 24 h. The mixtures were filtered and three 10.00 mL aliquots were taken for titration. The NaHCO_3_ and NaOH samples were acidified with 20.00 mL 0.05 M HCl, whereas for Na_2_CO_3_ samples 30.00 mL of 0.05 M HCl was added. The acidified solutions were then put into ultrasonic bath (Sonorex RK 52H, Bandelin electronic GmbH & Co. KG) for 20 min to expel dissolved CO_2_ and titrated with 0.05 M NaOH using phenolphthalein indicator.

## Results and discussion

### Hydrothermal treatment of brewer's spent grain

#### Effects of reaction temperature and time on adsorption capacity and chemical composition

**Fig. 1:**
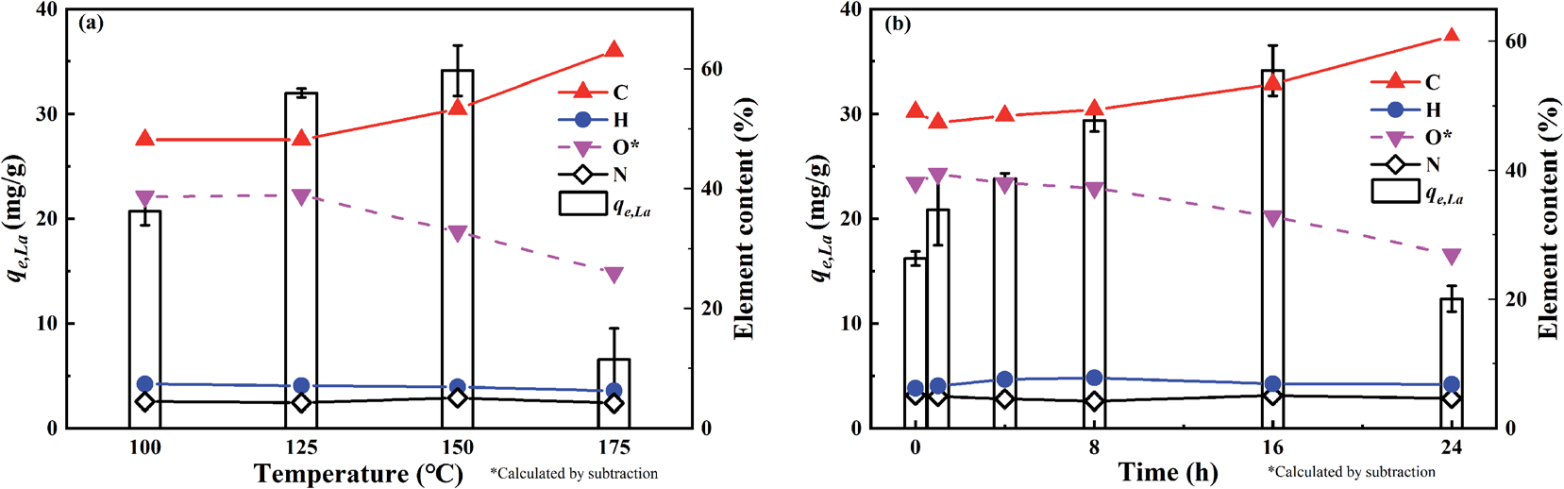
Effects of (a) reaction temperature (for 16 h) and (b) time (at 150 °C) on the adsorption capacity and chemical composition of ABSG. For adsorption experiments: 2 mg ABSG/2 mL solution, pH = 5.7, *c*_0_(La^3+^) = 100 mg L^−1^, *t* = 2 h, room temperature.

Temperature and time are two crucial factors for the hydrothermal process and the properties of the reaction product ABSG. The effects of both parameters on the adsorption capacity of La^3+^ and the chemical composition are illustrated in Fig. 1(a) and (b). As the temperature and time increase, the adsorption capacity for La^3+^ increases from 16.2 mg g^−1^ to 32.0 mg g^−1^ for a treatment at 125 °C for 16 h and reaches a maximum of 34.1 mg g^−1^ for a treatment at 150 °C for 16 h. The application of higher temperature and longer time of the hydrothermal treatment leads to a drop of the adsorption capacity to 6.5 mg g^−1^ (175 °C for 16 h). Therefore, the hydrothermal sample produced at 150 °C for 16 h (designated as ABSG) was chosen for the adsorption study and the general characterization (^13^C solid state NMR, oxygen functional groups and STA-MS-CS analysis).

**Fig. 2:**
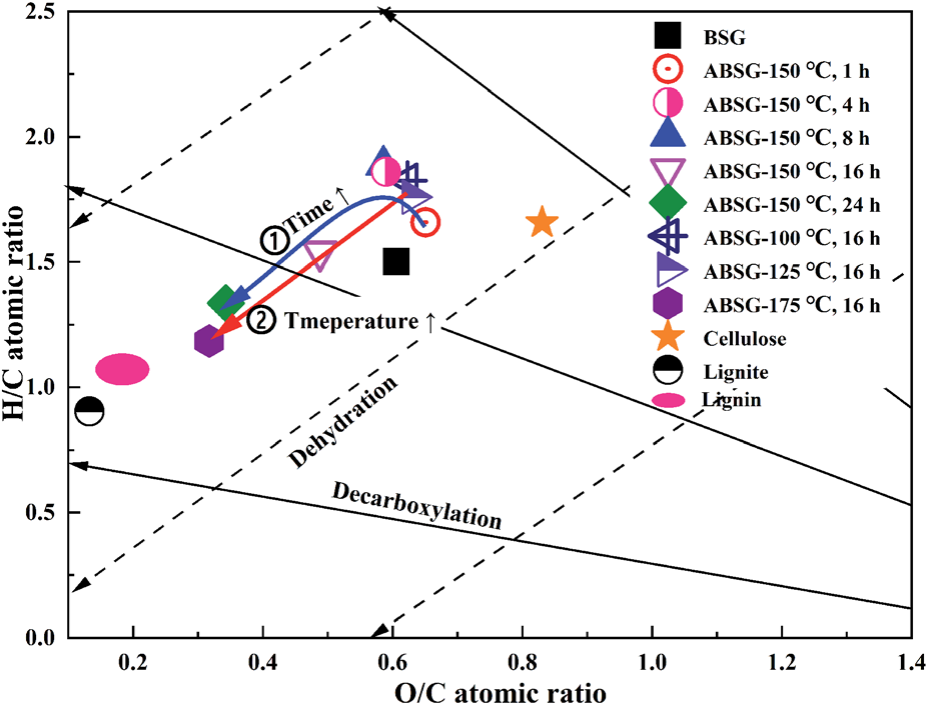
van Krevelen diagram of BSG-based biosorbents, typical biomass and coal.

Fig. 1(a) and (b) also show the changes of the element composition of ABSG as the reaction temperature and time varies. The carbon content increases as the reaction temperature increases and time prolongs, along with the decreasing of hydrogen and oxygen content. This may be due to the condensation polymerization mechanism which causes a loss of O-containing functional groups and low polarity of ABSG.^38^ This explains the decrease of adsorption capacity when the temperature and time exceed certain values. The nitrogen content of ABSG remains nearly unchanged as the temperature and time varies, which illustrates that N-containing groups have a higher persistence than O-containing functional groups. In this case, the N-containing groups may be incorporated into ABSG *via* Maillard reaction.^39^ In order to further investigate the reaction type during hydrothermal treatment, H/C atomic ratios *versus* O/C atomic ratios of different samples and reference materials are plotted in van Krevelen diagram^40^ as shown in Fig. 2. BSG is depicted as a kind of lignocellulose material with a chemical composition more similar to cellulose than pure lignin. The direct vector points at a dehydration pathway as the temperature increases, while both the dehydration and decarboxylation reaction occur with increasing reaction time. Both reaction pathways contribute to the lower H/C and O/C ratios of ABSG.^40^ In addition, the decrease of the H/C ratio also indicates aromatization mechanism during the hydrothermal conversion.^41^ It is noteworthy that all the reaction mechanisms, dehydration, decarboxylation, aromatization as well as Maillard reaction do not take place in consecutive steps, but rather in a parallel network of different pathways leading to the properties of ABSG.

#### Effects of reaction temperature and time on the surface functional groups

**Fig. 3:**
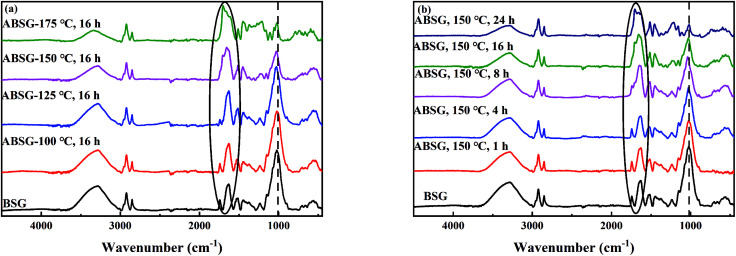
FT-IR spectra of BSG and ABSG samples with different (a) reaction temperature (for 16 h) and (b) time (at 150 °C). For adsorption experiments: 2 mg ABSG/2 mL solution, pH = 5.7, *c*_0_(La^3+^) = 100 mg L^−1^, *t* = 2 h, room temperature.

The FT-IR spectra provide important information of the surface functional groups on ABSG during the variation of temperature (Fig. 3(a)) and time (Fig. 3(b)). Basically, the chemical structure and functional groups of BSG remain unaffected during hydrothermal treatment. The absorption bands assigned to hydroxyl and amine groups vibration (3285 cm^−1^), –CH_2_– antisymmetric and symmetric stretching vibration (2924 cm^−1^, 2853 cm^−1^), amide I (1650 cm^−1^) and amide II (1518 cm^−1^) vibration, –COO^−^ symmetric stretching vibration (1453 cm^−1^), C–N stretching vibration (1245 cm^−1^) and C–O stretching vibration (1159 cm^−1^) in ester groups^42,43^ are observed for both BSG and the ABSG samples (see Fig. S2 in the ESI†). This demonstrates that the ABSG samples still preserve abundant number of functional groups. However, the intensity of –COOH groups stretching vibration at 1742 cm^−1^ gradually decreased as the temperature increases and time prolongs, which is probably due to the dehydration and condensation polymerization as discussed in the elemental analysis results. In addition, the intensity of the absorption band at 1650 cm^−1^ (C=O stretching vibration) increases when a temperature over 150 °C and a time over 8 h is applied, while the intensity of C–O stretching vibration at 1021 cm^−1^ decreases continuously with increasing temperature and time. These changes indicate the transformation of C–O bonds in the glucose units of cellulose and hemicellulose to C=O bonds,^41^ resulting in the increase of adsorption capacity when the temperature and time increase. When a temperature of 175 °C is applied, all above mentioned absorption bands decrease in intensity, indicating the loss of functional groups and thus decreasing adsorption capacity.

#### Effects of reaction temperature and time on Maillard reaction

**Fig. 4:**
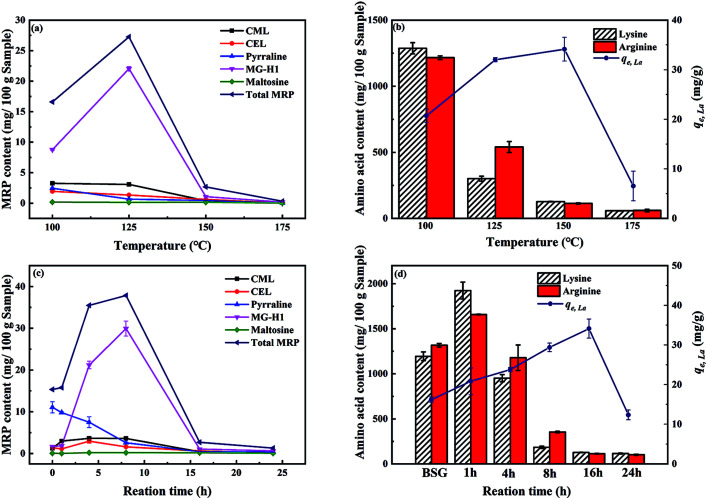
Effects of reaction temperature (for 16 h) on the (a) content of MRPs, (b) adsorption capacity and the content of amino acids, and effects of reaction time (at 150 °C) on the (c) content of MRPs, (d) adsorption capacity and the content of amino acids. For adsorption experiments: 2 mg ABSG/2 mL solution, pH = 5.7, *c*_0_(La^3+^) = 100 mg L^−1^, *t* = 2 h, room temperature.

It is well-known that Maillard reaction occurs when amino acids are mixed with reducing sugars at elevated temperature.^31^ As BSG is the exact mixture of proteins and lignocellulose, it is important to investigate the Maillard reaction for further understanding of the hydrothermal treatment. Fig. 4(a) illustrates the effect of reaction temperature on the content of typical Maillard reaction products (MRPs), namely *N*-ε-(carboxymethyl)lysine (CML), *N*-ε-(carboxyethyl)lysine (CEL), pyrraline, methylglyoxal-derived hydroimidazolone-1 (MG-H1) and maltosine as well as the total amount of Maillard reaction products (total MRP). The chemical structures of investigated MRPs are shown in Fig. S3 in the ESI.† The contents of two amino acids, lysine and arginine, which are mainly involved in the Maillard reaction^44^ depending on the treatment temperature are shown in Fig. 4(b). Of all studied MRPs, the highest content is observed for MG-H1 (up to 22 mg/100 g dry sample of ABSG-125 °C, 16 h, Fig. 4(a)), dominating their overall amount and dependency on the treatment temperature. The remaining MRPs are present in lower concentration (<5 mg/100 g dry sample) and follow in general the observed trend of MG-H1 (Fig. 4(a)). Nevertheless, since all MRPs may be involved in the metal adsorption, their total content is considered in the following discussion and analysis. When the treatment temperature increases from 100 °C to 125 °C the content of total MRPs increases. In addition, the content of lysine and arginine decreases, indicating that elevating the temperature promotes the Maillard reaction. After that the content of MRPs decreases as the temperature increases to 150 °C, while lysine and arginine are continuously consumed, indicating that the MRPs as well as the amino acids react further to form large, brown-black color polymeric compounds – melanoidins.^45^ The formation of melanoidins also explains the dark-brown color of ABSG (Fig. S4 in the ESI†) as carbonization of the BSG at the employed temperature is unlikely. Both, detected MRPs and melanoidins, are beneficial for metal ion complexation owing to their abundant functional groups and anionic charge.^45^ In addition, the reaction time shows a similar effect on the Maillard reaction as depicted in Fig. 4(c) and (d). Prolonging the reaction time promotes the Maillard reaction as amino acids are consumed and MRPs are generated. Consequently, the adsorption capacity of ABSG increases. Nevertheless, after 16 h of hydrothermal treatment, the contents of lysine and arginine decrease to a constant value, and further prolonging the reaction time results in a decrease of adsorption capacity, owing to the loss of functional groups.

### Characterization

**Fig. 5:**
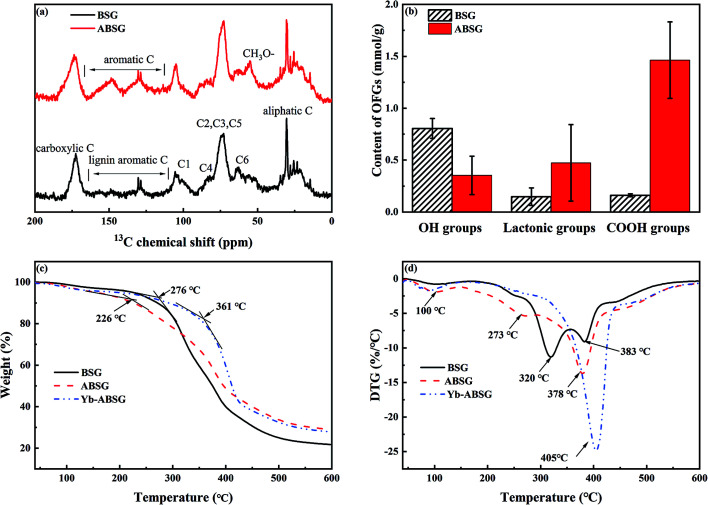
(a) ^13^C CP/MAS solid state NMR spectra of BSG and ABSG, (b) oxygen functional groups (OFGs) of BSG and ABSG, (c) TG and (d) DTG of BSG, ABSG and Yb-ABSG (20 °C min^−1^, He atmosphere).

In order to gain information about structure changes of BSG after hydrothermal treatment, ^13^C CP/MAS solid state NMR spectra (Fig. 5(a)) were recorded. The spectra show that after hydrothermal treatment the main chemical structure of BSG is conserved. The resonances in both spectra can be divided into four groups: aliphatic carbons (10–40 ppm), typical cellulose carbons C1 (105 ppm), C4 (80–90 ppm), C2,3,5 (72–75 ppm), C6 (62–65 ppm), lignin aromatic carbons (130 ppm) and carboxyl carbons (174 ppm).^46,47^ The emerging resonance between 110–160 ppm after hydrothermal treatment points at an increase content of unsaturated or aromatic carbons in ABSG. This is in accord with the increased intensity of the absorption band at 1650 cm^−1^ attributed to the stretching vibration of the C=O bond in the FT-IR spectra and the increase of the H/C ratio obtained in the elemental analysis. Compared to BSG, the ^13^C NMR spectrum of ABSG shows a more intense signal at 56 ppm due to methoxyl carbons, presumably bound to an aromatic ring,^47^ which also results from the aromatization during hydrothermal treatment. However, it must be noted that the quantitative analysis of CP spectra is limited by the different polarization transfer efficiencies of the various signals and depends on the water content of the sample. Probably, the extremely restricted mobility of the protons in BSG and/or their chemical inhomogeneity results in broad and hence practically invisible signals. In contrast, the slightly higher water content of ABSG may lead to better resolved signals in the spectrum. Note that a higher mobility of the protons can also cause a weakening or even suppression of the CP signals.

The content of the oxygen functional groups (OFG) of BSG and ABSG were quantified by Boehm titration. The results are depicted in Fig. 5(b) and show a decrease in the content of the hydroxyl groups for ABSG (0.48 mmol g^−1^) compared to BSG (0.8 mmol g^−1^). In contrast, the content of the lactonic groups increases from 0.15 mmol g^−1^ for BSG to 0.47 mmol g^−1^ for ABSG. The increase of the content of the carboxyl groups is even more pronounced from 0.15 mmol g^−1^ for BSG to 1.46 mmol g^−1^ for ABSG, which is comparable to the promoted oxidization of cellulosic materials (1.4 mmol g^−1^).^17^ However, this is rather unexpected as the decarboxylation and dehydration during the hydrothermal treatment of BSG would cause a loss of O-containing functional groups. Presumable three reasons can be assigned for this behavior: first, the emergence of carboxyl groups could be due to small fraction acids that form during the degradation of cellulose and hemicellulose, which are incorporated within the matrix as terminal groups.^48^ Second, the Maillard reaction produces various products with abundant functional groups, especially carboxyl groups. And third, it is possible that wet oxidation occurs producing carboxyl groups at the employed temperature (150 °C).^49^ The latter is supported by experiments employing an argon atmosphere during the hydrothermal treatment (150 °C, 16 h) yielding in an adsorbent (ABSG-Ar) with a reduced adsorption capacity (see Fig. S5 in the ESI†).

The thermal decomposition of BSG, ABSG and Yb^3+^ loaded ABSG (Yb-ABSG) was explored by STA-GC-MS analysis in order to obtain further information about the thermal stability and structural changes during the hydrothermal treatment and adsorption. Both BSG and ABSG show three derived thermogravimetric (DTG) peaks (maximum decomposition temperature) while Yb-ABSG shows only two peaks (Fig. 5(d)). The first DTG peak at around 100 °C could be assigned to the evaporation of water with a mass loss of 3.0 wt% (BSG), 4.3 wt% (ABSG) and 4.6 wt% (Yb-ABSG) in thermogravimetric curves (TG, Fig. 5(c)). The extrapolated onset temperatures in the TG of BSG, ABSG and Yb-ABSG are 276 °C, 226 °C and 361 °C, respectively. The lower of onset temperature after hydrothermal treatment is probably due to the increase of carboxyl groups on the surface of ABSG with low thermal stability.^50^ In contrast, an increase of the onset temperature to 361 °C is obtained for Yb-ABSG. Presumable, the coordination of the functional groups towards the metal ions may result in an increase in the thermal stability.^51^ The DTG-2 peaks could be attributed to the decomposition of hemicellulose and cellulose, while the DTG-3 peaks could be assigned to the continuous decomposition of cellulose materials and lignin.^52^ The DTG-3 peak area of ABSG is larger than that of BSG, which results from the degradation of intermediate hydrothermal products, *e.g.* advanced Maillard reaction products as well as polymerized melanoidins.^53^ For Yb-ABSG, DTG-2 and DTG-3 peaks overlap and form one large decomposition peak at higher temperature (405 °C). At even higher temperature, all samples undergo a moderate decomposition and carbonization stage with small change of sample mass. In this stage, some molecules in the hydrothermal samples (ABSG and Yb-ABSG) crosslink within the matrix,^54^ increasing the thermal stability and the residue mass from 21.7 wt% (BSG) to 29.0 wt% (ABSG) and 27.8 wt% (Yb-ABSG).

The volatile products at 375–385 °C (BSG, ABSG) and at 340–360 °C (Yb-ABSG) are analyzed by GC-MS (Table S4 in the ESI†). CO_2_ and water are the most abundant products, of which CO_2_ counting for 67.7% (BSG), 48.6% (ABSG) and 42.1% (Yb-ABSG) and water for 25.4% (BSG), 43.8% (ABSG) and 39.7% (Yb-ABSG) of the total analyzed products. The release of CO_2_ results from the decomposition of O-functional groups, while the source of water could be ascribed to the dehydration and fragmentation of the hydroxyl substituents in lignin, hemicellulose and cellulose.^55^ A group of furan-derivatives *e.g.* 3-methylfuran and 2,5-dimethylfuran are detected with over 0.9% among the products, and small molecules such as acetic anhydride and 1-hydroxypropan-2-one are also noticed. These products are evolved from cellulose degradation under helium atmosphere.^42^ It is noteworthy that acetic acid is not detected during the decomposition of BSG. However, approximately 1–3% among the decomposition products of ABSG (1.3%) and Yb-ABSG (2.7%) are detected as acetic acid. This increase is in agreement with the abovementioned enhanced presence of carboxyl groups on the surface of ABSG.

### Adsorption study

The preliminary adsorption experiments employing La^3+^ show that the highest adsorption of 34.1 mg g^−1^ is obtained for ABSG treated at 150 °C and 16 h (see Fig. 1(a) and (b)). Thus, the biosorbents BSG and ABSG (150 °C, 16 h) were used for the batch adsorption experiments using UO_2_^2+^, La^3+^, Eu^3+^ and Yb^3+^. ICP-OES was used to determine the concentration of the metal ions before and after the adsorption. In addition, adsorption experiments were also performed using the radiotracer technique employing the radiation from ^169^Yb. The results are depicted in Fig. S6 in the ESI† and show comparable adsorption capacities for BSG (21 mg g^−1^ for ICP method and 22 mg g^−1^ for radiotracer method) and ABSG (42 mg g^−1^ for ICP method and 49 mg g^−1^ for radiotracer method), indicating both methods can be used.

#### Effect of initial pH

**Fig. 6:**
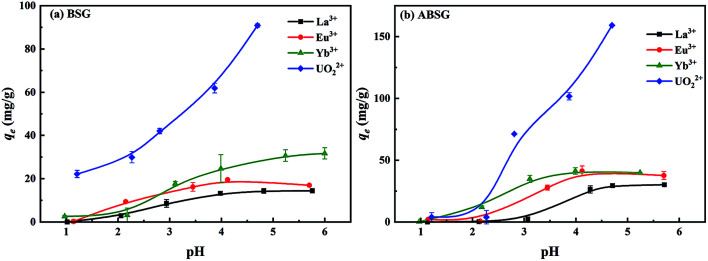
Effect of initial pH on the adsorption capacity of (a) BSG and (b) ABSG. For adsorption: 2 mg adsorbent/2 mL solution, *c*_0_(La^3+^, Eu^3+^, Yb^3+^) = 100 mg L^−1^, *c*_0_(UO_2_^2+^) = 300 mg L^−1^, 2 h, room temperature.

The initial pH of the target aqueous phase is crucial for the adsorption process as it could influence both the surface properties of adsorbents and the species of adsorbates. Fig. 6(a) and (b) depict the effect of initial pH on the adsorption capacity of BSG and ABSG respectively, and the determined pH_pzc_ of both adsorbents are shown in Fig. S7 in the ESI.† The pH_pzc_ of ABSG is 4.1, which is lower than that of BSG (pH_pzc_ = 5.7). This may be due to the increased number of carboxyl groups causing a more negative charged surface of ABSG. When the initial pH of adsorption is lower than the pH_pzc_ of the adsorbent, the surface charge of adsorbent would be positive. Thus, the functional groups are protonated, which is unfavorable for the metal ion adsorption considering the electrostatic repulsive effect.^56^ Furthermore, large amount of H^+^ in low pH solution would generate great competitive effect towards metal ions, hindering the adsorption process.^27^ In agreement to these effects a low adsorption capacity is obtained for all studied metal ions for a pH ranging from 1 to 3 (Fig. 6(a) and (b)). As the initial pH increases over the pH_pzc_, the functional groups are deprotonated, thus the adsorbent surface becomes negative, and electrostatic attraction towards the adsorbates increases. As a consequence, the adsorption of La^3+^, Eu^3+^ and Yb^3+^ increases as the pH increases, and gradually reaches saturated adsorption capacity at the pH between 5 and 6. Nevertheless, the adsorption capacity of UO_2_^2+^ increases without plateau. This may be attributed to the change of uranyl species. When the pH rises from 3 to 5, hydrated species like (UO_2_)_2_(OH)_2_^2+^, UO_2_OH^+^ and (UO_2_)_3_(OH)^5+^ occur with UO_2_^2+^.^57^ These hydrated species are more easily adsorbed due to their high affinity for solid surfaces.^58^

#### Kinetic study

**Fig. 7:**
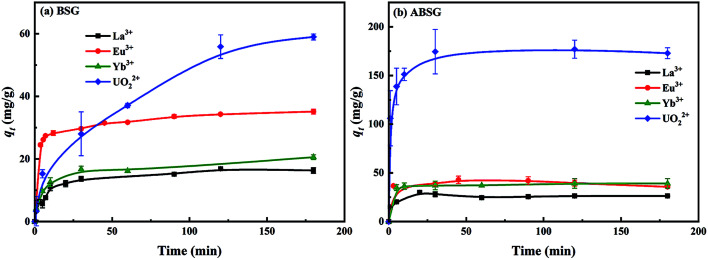
Effect of contact time on the adsorption capacity of (a) BSG and (b) ABSG. For adsorption, 2 mg adsorbent/2 mL solution, *c*_0_(La^3+^, Eu^3+^, Yb^3+^) = 100 mg L^−1^, *c*_0_(UO_2_^2+^) = 300 mg L^−1^, pH(La^3+^, Eu^3+^, Yb^3+^) = 5.7, pH(UO_2_^2+^) = 4.7, room temperature.

The results of adsorption experiments in dependent of the contact time for BSG and ABSG are depicted in Fig. 7(a) and (b). The obtained results are fitted using pseudo-first-order kinetic (PFO) model and pseudo-second-order kinetic (PSO) model as shown in Table 1.^59^ For BSG, the equilibrium time increases as the adsorption capacity of studied metal ions increases, especially in the case of UO_2_^2+^, 2 h is required to complete the adsorption process. In contrast, rapid adsorption is observed in the first 30 minutes for of all four ions employing ABSG and the equilibrium is reached after 1 h. This behavior indicates that adsorption occurs mainly on the outer surface of ABSG with large amounts of adsorption sites.^59^ For both adsorbents, the correlation coefficients (*R*^2^) of PSO model are higher than those of PFO model, and the calculated equilibrium adsorption capacity (*q*_e,cal_, mg g^−1^) agrees to a larger extend to the experimental equilibrium adsorption capacity (*q*_e,exp_, mg g^−1^). For example, the *R*^2^ using the PSO model to fit the UO_2_^2+^ onto ABSG adsorption data is 0.9996, which is higher than the *R*^2^ of the PFO model (0.9955). In addition, the *q*_e,cal_ of the PSO model (175.1 mg g^−1^) is in good agreement with the experimental *q*_e,exp_ = 177.0 mg g^−1^ compared to a *q*_e,cal_ = 75.2 mg g^−1^ for the PFO model (see Table 1). Thus, the PSO model could better describe and predict the adsorption behavior of ABSG, and it could be referred that the rate control step of the adsorption process is chemisorption.^25^

**Table 1:** Kinetic fitting results and parameters of BSG and ABSG. For adsorption, 2 mg adsorbent/2 mL solution, *c*_0_(La^3+^, Eu^3+^, Yb^3+^) = 100 mg L^−1^, *c*_0_(UO_2_^2+^) = 300 mg L^−1^, pH(La^3+^, Eu^3+^, Yb^3+^) = 5.7, pH(UO_2_^2+^) = 4.7, room temperature

Adsorbent	Adsorbate	Kinetic model	*R* ^2^	*k* (g (mg^−1^ min^−1^))	*q*_e,cal_ (mg g^−1^)	*q*_e,exp_ (mg g^−1^)
BSG	La^3+^	PFO	0.3078	0.0011	4.1	16.9
		PSO	0.9982	0.0055	16.5	
	Eu^3+^	PFO	0.9835	0.0203	9.8	35.1
		PSO	0.9987	0.0032	35.4	
	Yb^3+^	PFO	0.5726	0.0206	11.6	20.6
		PSO	0.9988	0.0140	20.9	
	UO_2_^2+^	PFO	0.9439	0.0230	59.2	59.0
		PSO	0.9604	0.0140	67.0	
ABSG	La^3+^	PFO	0.9510	0.0267	9.0	30.0
		PSO	0.9968	0.7062	26.0	
	Eu^3+^	PFO	0.8576	0.0158	4.1	40.2
		PSO	0.9986	0.0213	36.9	
	Yb^3+^	PFO	0.6214	0.0074	2.8	39.2
		PSO	0.9983	0.0181	39.2	
	UO_2_^2+^	PFO	0.9955	0.1126	75.2	177.0
		PSO	0.9996	0.0077	175.1	

#### Isotherms study

**Fig. 8:**
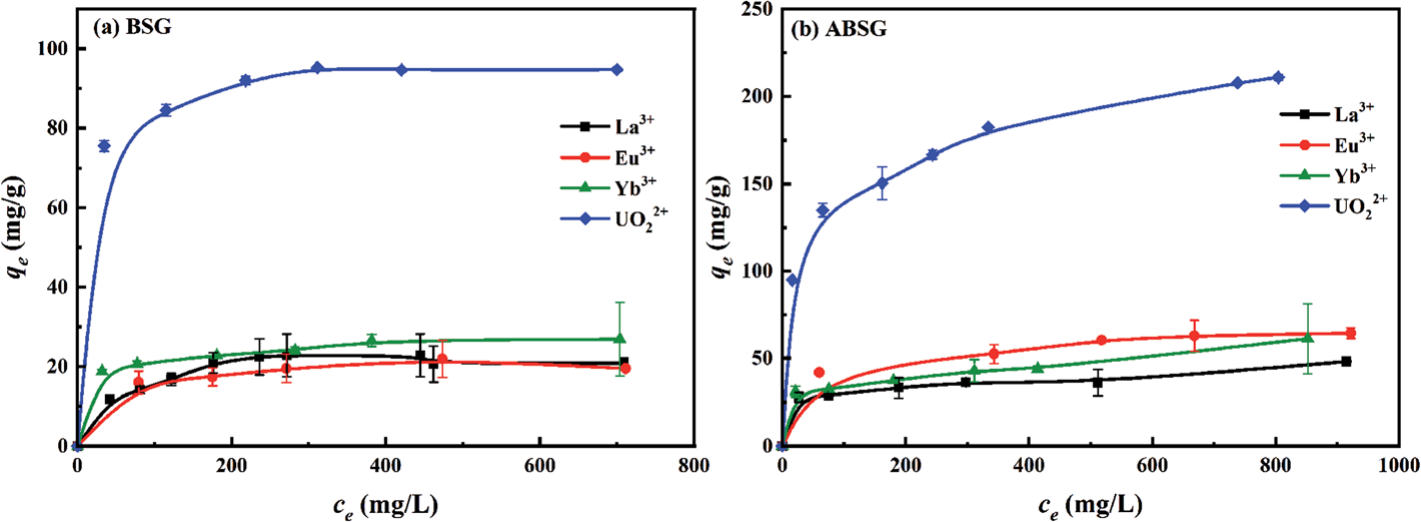
Effect of initial concentration on the adsorption capacity of (a) BSG and (b) ABSG. For adsorption, 2 mg adsorbent/2 mL solution, *t*_(BSG)_ = 2 h, *t*_(ABSG)_ = 1 h, pH(La^3+^, Eu^3+^, Yb^3+^) = 5.7, pH(UO_2_^2+^) = 4.7, room temperature.

Investigation of the equilibrium relationships of the adsorption process, namely the isotherms, is essential to understand the adsorption process and quantitatively compare the adsorption capacity in different adsorption systems.^60^Fig. 8(a) and (b) illustrate the adsorption isotherms of La^3+^, Eu^3+^, Yb^3+^ and UO_2_^2+^ onto BSG and ABSG, and the fitting results of Langmuir and Freundlich model are summarized in Table 2. Graphically the adsorption isotherms are featured by the increase adsorption capacity along with the increase of initial concentration, and then followed with a plateau, which is typical for Langmuir type adsorption.^61^ In addition, employing the Langmuir model gives better prediction of the adsorption as represented by the high correlation coefficients of *R*^2^ > 0.98 for all studied adsorption processes. Langmuir isotherm model is widely applied to evaluate the performance of different biosorbents,^12^ and assumes that monolayer adsorption occurs with identical and equivalent adsorption sites on the surface of adsorbents.^60^ Furthermore, the separation factor (*R*_L_) defined by Weber and Chakravorti^62^ reflects the adsorption nature of the studied ions. In all cases, the *R*_L_ is in the range of 0 to 1 (Table 2), which indicates a favorable adsorption process. The maximum adsorption capacity of La^3+^, Eu^3+^, Yb^3+^ and UO_2_^2+^ onto ABSG are calculated as 37.5 mg g^−1^, 68.3 mg g^−1^, 46.0 mg g^−1^ and 220.6 mg g^−1^, respectively, which is an increase by 27%, 172%, 65% and 130% compared to BSG (La^3+^ 29.4 mg g^−1^, Eu^3+^ 25.1 mg g^−1^, Yb^3+^ 27.8 mg g^−1^ and UO_2_^2+^ 96.0 mg g^−1^). The obtained order of a preferred adsorption of Eu^3+^ over Yb^3+^ and La^3+^ is observed previously in the studies of using immobilized *Pseudomonas aeruginosa* as biosorbent^16^ and may reflect the variation in the binding mode of metal in the adsorption process (see section of adsorption mechanism). Nevertheless, the adsorption capacity of ABSG is comparable to typical biosorbents for the studied ions with fast kinetics, like ultrafine cellulose nanofibers (167 mg g^−1^ for UO_2_^2+^),^17^ pectin (41.2 mg g^−1^ for La^3+^)^63^ and yeast embedded cellulose (25.9 mg g^−1^ for Eu^3+^)^15^ (see Fig. S8 in the ESI†). The obtained adsorption capacity of ABSG for the selected rare earth metal ions is also higher than that of graphene oxide–melamine composites (25.04 mg g^−1^ for La^3+^, 26.68 mg g^−1^ for Eu^3+^ and 20.88 mg g^−1^ for Yb^3+^)^64^ and graphene oxide–tris(4-aminophenyl)amine composites (10.52 mg g^−1^ for La^3+^, 30.88 mg g^−1^ for Yb^3+^)^8^ reported in the literature. Although some adsorbents like elastic diglycolamic-acid modified chitosan sponges (CSs-DGAA)^65^ and graphene oxide-activated carbon felt composite^18^ show higher adsorption capacity in case of Eu^3+^ (79 mg g^−1^) and UO_2_^2+^ (298 mg g^−1^), it should be taken into consideration that the preparation of synthetic adsorbents is often costly, complicated and environmentally unsustainable. On the contrary, the preparation of ABSG is simple and environmental benign without harmful chemicals and complicated process. The energy consumption has been minimized by applying a temperature that is 70 °C lower than HTC. Both BSG and ABSG do not exhibit obvious selectivity among the rare earth metal ions, except for the adsorption preference towards uranyl ions with a high adsorption capacity. However, this could also be justified that ABSG could simultaneously remove the hazardous uranyl and rare earth metal ions from the wastewater, which simplify the treatment procedure and reduce the cost.

**Table 2:** Isotherms fitting results and parameters of BSG and ABSG. For adsorption, 2 mg adsorbent/2 mL solution, *t*_(BSG)_ = 2 h, *t*_(ABSG)_ = 1 h, pH(La^3+^, Eu^3+^, Yb^3+^) = 5.7, pH(UO_2_^2+^) = 4.7, room temperature

Adsorbate	Adsorbent	Langmuir model			Freundlich model	
		*R* ^2^	*q*_m_ (mg g^−1^)	*R*_L_	*R* ^2^	*n*
La^3+^	BSG	0.9823	29.4	0.2073–0.6108	0.9775	2.60
	ABSG	0.9981	37.4	0.0837–0.4773	0.8975	9.74
Eu^3+^	BSG	0.9981	25.1	0.0630–0.4022	0.9738	6.03
	ABSG	0.9926	68.3	0.0596–0.3880	0.9809	6.12
Yb^3+^	BSG	0.9972	27.8	0.0286–0.5706	0.9631	8.00
	ABSG	0.9930	46.0	0.0258–0.3164	0.8654	8.03
UO_2_^2+^	BSG	0.9997	96.0	0.0092–0.0915	0.9907	9.42
	ABSG	0.9914	220.6	0.0541–0.5411	0.9872	4.97

#### Thermodynamics study

The thermodynamic analysis was performed by recording adsorption isotherms of uranyl and rare earth metal ions for BSG and ABSG at different temperatures (Fig. S9, ESI†), a linear fitting of the isotherms using Langmuir model (Fig. S10, ESI†) and a linearized plot of ln *K*^0^_e_*versus* 1/*T* for thermodynamic parameter calculations (Fig. S11, ESI†) are given in the ESI.†Table 3 summarizes the thermodynamic parameters of the different adsorption process. The negative values of Δ*G*^0^ of all studied cases indicate a spontaneous adsorption process. In addition, the positive values of Δ*S*^0^ in all cases could be explained as the increase of randomness at the solid/liquid interface during the adsorption.^66^ Comparing the Δ*H*^0^ values with −*T*Δ*S*^0^ values shows that the thermodynamic driving force of the uranyl and rare earth metal ions onto the biosorbents is the change of entropy.^67^ The adsorption of the studied rare earth metal ions onto ABSG and BSG presents positive Δ*H*^0^, indicating an endothermic process as reported in most of the literature.^68^ The adsorption of UO_2_^2+^ onto BSG is also endothermic (Δ*H*^0^ = 55.8 kJ mol^−1^), which is consistent with the uranyl complexation by anionic organic ligands, producing endothermic enthalpies and large positive entropies of complexation.^18^ Nevertheless, the adsorption of UO_2_^2+^ onto ABSG is exothermic (Δ*H*^0^ = −12.6 kJ mol^−1^). Literature has reported that different surface properties of biochar would result in different thermodynamic mechanisms of uranyl adsorption.^67^ Therefore, variations of the surface characters of BSG and ABSG such as the content of carboxyl groups (0.15 mmol g^−1^ for BSG and 1.46 mmol g^−1^ for ABSG) and pH_PZC_ (5.7 for BSG and 4.1 for ABSG) may result in the obtained difference in Δ*H*^0^.

**Table 3:** Thermodynamic parameters of uranyl and rare earth metal ions adsorption onto BSG and ABSG. For adsorption, 2 mg adsorbent/2 mL metal solution, *c*_0_ = 100–600 mg L^−1^, *t*_BSG_ = 2 h, *t*_ABSG_ = 1 h, pH(UO_2_^2+^) = 4.7, pH(La^3+^, Eu^3+^, Yb^3+^) = 5.7, *T* = 25 °C, 45 °C, 65 °C, stirrer speed = 180 rpm

Adsorbent	Adsorbate	Δ*H*^0^ (kJ mol^−1^)	Δ*S*^0^ (J (mol^−1^ K^−1^))	Δ*G*^0^ (kJ mol^−1^)			−*T*Δ*S*^0^ (kJ mol^−1^)		
				298 K	318 K	338 K	298 K	318 K	338 K
ABSG	UO_2_^2+^	−12.6	39.6	−24.4	−25.2	−25.9	−11.8	−12.6	−13.4
	Yb^3+^	14.4	113.1	−19.3	−21.5	−23.8	−33.7	−36.0	−38.2
	Eu^3+^	18.0	124.3	−19.1	−21.6	−24.0	−37.0	−39.5	−42.0
	La^3+^	12.2	109.5	−20.4	−22.6	−24.8	−32.6	−34.8	−37.0
BSG	UO_2_^2+^	55.8	261.1	−22.0	−27.2	−32.5	−77.8	−83.0	−88.3
	Yb^3+^	27.0	146.3	−16.6	−19.5	−22.4	−43.6	−46.5	−49.4
	Eu^3+^	12.6	108.1	−19.6	−21.8	−24.0	−32.2	−34.4	−36.5
	La^3+^	7.5	89.8	−19.3	−21.1	−22.9	−26.8	−28.6	−30.4

### Investigation of adsorption mechanism

The determined BET surface area of BSG and ABSG is <2 m^2^ g^−1^, which is consistent with the literature reporting a BET surface of BSG of 0.48 m^2^ g^−1^.^69^ Thus, the surface area of the biosorbent is very small and has little effect on the adsorption process.

**Fig. 9:**
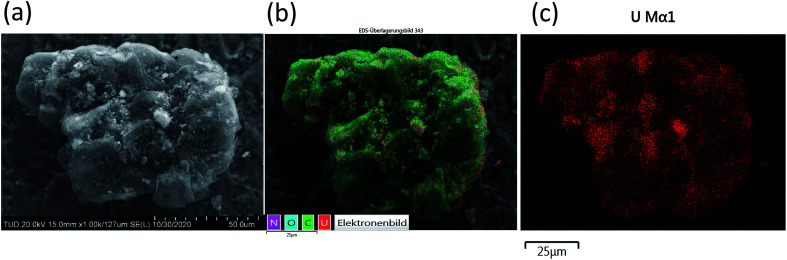
(a) SEM image (magnification of 1000 times) of uranyl ions loaded-ABSG, (b) EDX element mapping of uranyl ions loaded-ABSG (20 kV/10 μA, magnification of 1000 times, 25 frames) and (c) distribution of uranium on uranyl ions loaded-ABSG. For ion-loading: 50 mg ABSG/50 mL solution, pH(UO_2_^2+^) = 4.7, *c*_0_(UO_2_^2+^) = 300 mg L^−1^, *t* = 1 h, room temperature.

SEM/EDX analysis prove the metal ion adsorption onto to surface of ABSG, as the SEM image, the EDX element mapping and the uranium distribution of uranyl ions-loaded ABSG depicted in Fig. 9 indicate. Similar results are obtained for the examined rare earth metal ions and are displayed in Fig. S12 in the ESI.† The SEM images show an irregular and rough surface of ABSG with no apparent pore structure (Fig. 9(a)), which is consistent with the low BET surface area. Therefore, the adsorption of the metal ions by ABSG is due to interactions of the functional groups on the surface. This is supported by the EDX element mapping (Fig. 9(b)) and the distribution of uranium (Fig. 9(c)). The irregular shape of the particles leads to noticeable shading so that no uniform recording of the X-ray signal can be expected. However, besides some small areas with uranium accumulation the uranyl ions seem to be more or less evenly distributed on the surface of ABSG.

**Fig. 10:**
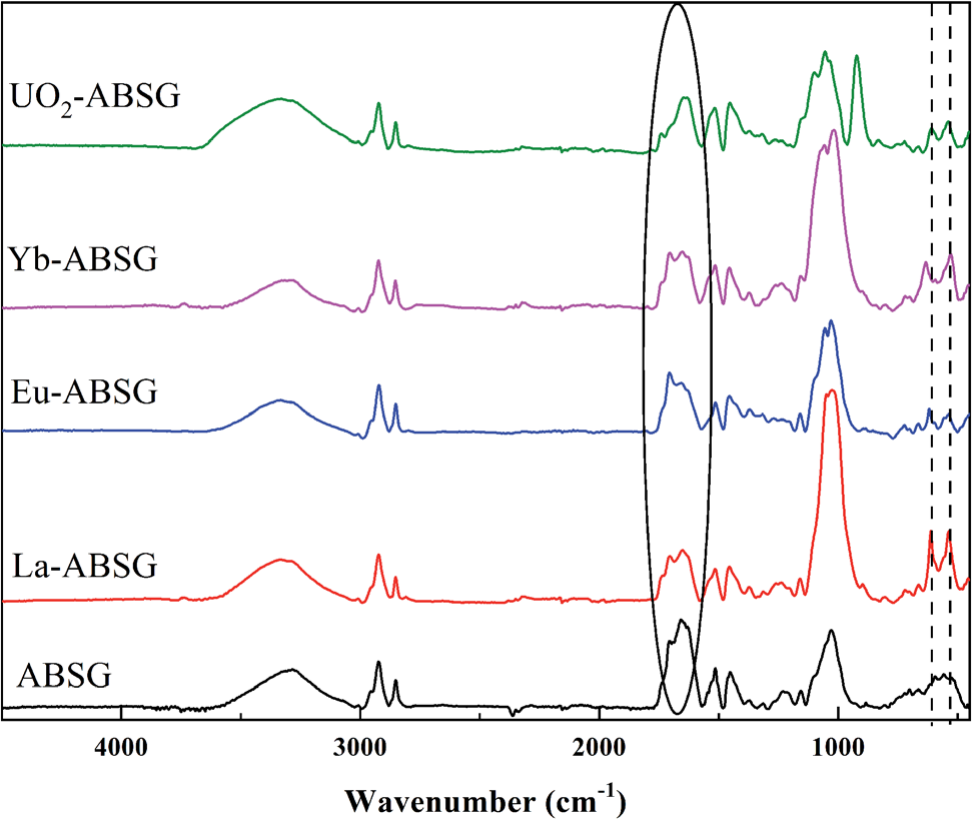
FT-IR spectra of ABSG and ion-loaded ABSG. For ion-loading: 50 mg ABSG/50 mL solution, pH(La^3+^, Eu^3+^, Yb^3+^) = 5.7, pH(UO_2_^2+^) = 4.7, *c*_0_(La^3+^, Eu^3+^, Yb^3+^) = 100 mg L^−1^, *c*_0_(UO_2_^2+^) = 300 mg L^−1^, *t* = 1 h, room temperature.

FT-IR spectra of ABSG before and after adsorption are displayed in Fig. 10. For all ion-loaded samples the strengths of the absorption bands at 1654 cm^−1^ are decreased, which could be attributed to the antisymmetric stretching vibration of –COO^−^ groups. This indicates the involvement of carboxyl groups in the adsorption process. In addition, the chelate model is calculated based on the differences between the antisymmetric and symmetric stretching bands of the –COO^−^ groups (Δ_*ν*_as_–*ν*_s__). As shown in Table 4, Δ_*ν*_as_–*ν*_s__ values of 197 cm^−1^ and 201 cm^−1^ are obtained for La^3+^ and Yb^3+^ loaded samples, respectively. This minor difference is negligibly small considering the resolution of the recorded spectra of 4 cm^−1^. In general, value of Δ_*ν*_as_–*ν*_s__ > 200 cm^−1^ indicates a monodentate binding of metal ions, which involves an interaction with the metal ions through an electrostatic effect and ion exchange.^70^ In contrast, Δ_*ν*_as_–*ν*_s__ of 179 cm^−1^ and 187 cm^−1^ are obtained for the Eu^3+^ and UO_2_^2+^ loaded samples, pointing at a different binding mode. In the case of UO_2_^2+^, this is presumable due to the presence of hydrated uranyl species and a more complex coordination occurs. This is confirmed by a new absorption band occurring at 922 cm^−1^ which is assigned to the antisymmetric stretching vibration of U=O in (UO_2_)_3_(OH)^5+^.^71^ In addition, new absorption bands are obtained for all studied metal ions after adsorption at 530 to 540 cm^−1^ and 440 to 450 cm^−1^, which could be ascribed to metal–N vibration and metal–O vibration, respectively.^72^ This indicates that N-containing functional groups of the ABSG also contribute to the adsorption of the studied ions. And is consistent with the literature reporting the involvement of O and N-containing functional groups for the adsorption of uranyl^73^ and rare earth metal ions.^8^ Thus, the adsorption of La^3+^, Eu^3+^, Yb^3+^ and UO_2_^2+^ onto ABSG is predominated by the electrostatic effect between negative charged functional groups and cations. However, in case of Eu^3+^ and UO_2_^2+^, a coordination of the cation with O and N-containing functional groups also plays an important role, thus resulting in higher adsorption capacity than that of La^3+^ and Yb^3+^ as observed in the isotherms study.

**Table 4:** Calculation results of the FT-IR data

	ABSG	La-ABSG	Eu-ABSG	Yb-ABSG	UO_2_-ABSG
Antisymmetric stretching of –COO^−^ (*ν*_as_, _−COO_^−^), cm^−1^	1654	1650	1631	1655	1639
Symmetric stretching of –COO^−^ (*ν*_s_, _−COO_^−^), cm^−1^	1452	1453	1453	1454	1452
Δ_*ν*_as_–*ν*_s__, cm^−1^	202	197	179	201	187

## Conclusions

In present study, BSG is successfully transformed into an effective biosorbent ABSG *via* a mild hydrothermal treatment approach (150 °C, 16 h) in line with the principles of green chemistry. This is performed at a significantly lower temperature than traditional HTC method without additional activation process, which minimizes the energy consumption and environmental impact during the treatment. Maillard reaction with the formation of melanoidins plays an important role in the increase of adsorption capacity, along with other pathways like dehydration, decarboxylation, aromatization and oxidation. Thus, the ABSG obtained has an increase content of carboxyl groups from 0.15 mmol g^−1^ (BSG) to 1.46 mmol g^−1^ with increasing adsorption capacity. The Langmuir maximum adsorption capacity of La^3+^, Yb^3+^, Eu^3+^ and UO_2_^2+^ onto ABSG obtained at optimal pH of 5.7 for La^3+^, Eu^3+^ and Yb^3+^ and at 4.7 for UO_2_^2+^ is 38, 46, 68 and 221 mg g^−1^, respectively. In addition, FT-IR spectra show that both O-containing functional groups and N-containing functional groups are involved in the adsorption of studied ions. Overall, the present study demonstrates the potential of hydrothermal treated BSG for uranyl and rare earth metal ions removal from wastewater with low cost and high sustainability. Furthermore, the results about Maillard reaction provide information regarding the hydrothermal treatment of protein-rich biomass at low temperature, which may give inspirations on the exploration of similar biomass as biosorbents after treatment at low temperatures.

## Conflicts of interest

There are no conflicts to declare.

## Supplementary Material

RA-010-D0RA08164G-s001

## References

[cit1] Waseem A., Ullah H., Rauf M. K., Ahmad I. (2015). Crit. Rev. Environ. Sci. Technol..

[cit2] Brugge D., Buchner V. (2011). Rev. Environ. Health.

[cit3] Herschy R. W. , in Encyclopedia of Lakes and Reservoirs, ed. L. Bengtsson, R. W. Herschy and R. W. Fairbridge, Springer Netherlands, Dordrecht, 2012, pp. 876–883

[cit4] Veliscek-Carolan J. (2016). J. Hazard. Mater..

[cit5] Tunsu C., Petranikova M., Gergorić M., Ekberg C., Retegan T. (2015). Hydrometallurgy.

[cit6] Pagano G., Guida M., Tommasi F., Oral R. (2015). Ecotoxicol. Environ. Saf..

[cit7] Liao C., Zhao X. R., Jiang X. Y., Teng J., Yu J. G. (2020). Microchem. J..

[cit8] Zhao X., Jiang X., Peng D., Teng J., Yu J. (2021). J. Rare Earths.

[cit9] Kegl T., Košak A., Lobnik A., Novak Z., Kralj A. K., Ban I. (2020). J. Hazard. Mater..

[cit10] Kumari A., Singh S., Parmar K., Pathak D. D., Kumar Jha M. (2020). J. Ind. Eng. Chem..

[cit11] Takei T., Takehara M., Takabayashi T., Yanagida S., Kumada N. (2020). Colloids Surf., A.

[cit12] Gupta N. K., Sengupta A., Gupta A., Sonawane J. R., Sahoo H. (2018). J. Environ. Chem. Eng..

[cit13] Rajasulochana P., Preethy V. (2016). Resour. Technol..

[cit14] Nicomel N. R., Otero-Gonzalez L., Arashiro L., Garfí M., Ferrer I., Van Der Voort P., Verbeken K., Hennebel T., Du Laing G. (2020). Green Chem..

[cit15] Arunraj B., Sathvika T., Rajesh V., Rajesh N. (2019). ACS Omega.

[cit16] Texier A. C., Andrès Y., Faur-Brasquet C., Le Cloirec P. (2002). Chemosphere.

[cit17] Ma H., Hsiao B. S., Chu B. (2012). ACS Macro Lett..

[cit18] Chen S., Hong J., Yang H., Yang J. (2013). J. Environ. Radioact..

[cit19] Nicolae S. A., Au H., Modugno P., Luo H., Szego A. E., Qiao M., Li L., Yin W., Heeres H. J., Berge N., Titirici M.-M. (2020). Green Chem..

[cit20] Heidari M., Dutta A., Acharya B., Mahmud S. (2019). J. Energy Inst..

[cit21] Zhang Z., Zhu Z., Shen B., Liu L. (2019). Energy.

[cit22] Zhang Z. B., Cao X. H., Liang P., Liu Y. H. (2013). J. Radioanal. Nucl. Chem..

[cit23] Cai H., Lin X., Tian L., Luo X. (2016). Ind. Eng. Chem. Res..

[cit24] Kambo H. S., Dutta A. (2015). Renewable Sustainable Energy Rev..

[cit25] Lu S., Gibb S. W. (2008). Bioresour. Technol..

[cit26] Low K. S., Lee C. K., Low C. H. (2001). J. Appl. Polym. Sci..

[cit27] Wierzba S., Kłos A. (2019). J. Cleaner Prod..

[cit28] Wierzba S., Rajfur M., Nabrdalik M., Kłos A. (2019). Microchem. J..

[cit29] de Araújo T. P., Quesada H. B., Bergamasco R., Vareschini D. T., de Barros M. A. S. D. (2020). Bioresour. Technol..

[cit30] Mussatto S. I., Dragone G., Roberto I. C. (2006). J. Cereal Sci..

[cit31] Hellwig M., Henle T. (2014). Angew. Chem., Int. Ed..

[cit32] Kegl T., Ban I., Lobnik A., Košak A. (2019). J. Hazard. Mater..

[cit33] Merrill A. L. and Watt B. K., Energy value of foods: basis and derivation, ARS United States Department of Agriculture, Washington DC, 1973

[cit34] Schwarzenbolz U., Hofmann T., Sparmann N., Henle T. (2016). J. Agric. Food Chem..

[cit35] Hellwig M., Witte S., Henle T. (2016). J. Agric. Food Chem..

[cit36] Balistrierl L. S., Murray J. W. (1981). Am. J. Sci..

[cit37] Oickle A. M., Goertzen S. L., Hopper K. R., Abdalla Y. O., Andreas H. A. (2010). Carbon.

[cit38] Funke A., Ziegler F. (2010). Biofuels, Bioprod. Biorefin..

[cit39] Arauzo P. J., Du L., Olszewski M. P., Meza Zavala M. F., Alhnidi M. J., Kruse A. (2019). Bioresour. Technol..

[cit40] Xu X., Tu R., Sun Y., Wu Y., Jiang E., Zhen J. (2019). Bioresour. Technol..

[cit41] Simsir H., Eltugral N., Karagoz S. (2017). Bioresour. Technol..

[cit42] Balogun A. O., Sotoudehniakarani F., McDonald A. G. (2017). J. Anal. Appl. Pyrolysis.

[cit43] Li Q., Chai L., Wang Q., Yang Z., Yan H., Wang Y. (2010). Bioresour. Technol..

[cit44] Lund M. N., Ray C. A. (2017). J. Agric. Food Chem..

[cit45] Rufián-Henares J. A., De La Cueva S. P. (2009). J. Agric. Food Chem..

[cit46] Li Y., Cui D., Tong Y., Xu L. (2013). Int. J. Biol. Macromol..

[cit47] Weber B., Stadlbauer E. A., Eichenauer S., Koch C., Albert K., Kramer M., Steffens D. (2013). J. Biobased Mater. Bioenergy.

[cit48] Qi X., Li L., Tan T., Chen W., Smith R. L. (2013). Environ. Sci. Technol..

[cit49] Yousefifar A., Baroutian S., Farid M. M., Gapes D. J., Young B. R. (2017). Bioresour. Technol..

[cit50] Dos Santos D. M., De Lacerda Bukzem A., Ascheri D. P. R., Signini R., De Aquino G. L. B. (2015). Carbohydr. Polym..

[cit51] Hardy A., Vanhoyland G., Geuzens E., Van Bael M. K., Mullens J., Van Poucke L. C., D'Haen J. (2005). J. Sol-Gel Sci. Technol..

[cit52] Osman A. I., O'Connor E., McSpadden G., Abu-Dahrieh J. K., Farrell C., Al-Muhtaseb A. H., Harrison J., Rooney D. W. (2020). J. Chem. Technol. Biotechnol..

[cit53] Kruse A., Zevaco T. (2018). Energies.

[cit54] Olszewski M. P., Arauzo P. J., Maziarka P. A., Ronsse F., Kruse A. (2019). Catalysts.

[cit55] Özsin G., Pütün A. E. (2017). Energy Convers. Manage..

[cit56] Qu J., Meng X., Zhang Y., Meng Q., Tao Y., Hu Q., Jiang X., You H., Shoemaker C. A. (2019). J. Hazard. Mater..

[cit57] Misaelides P., Godelitsas A., Filippidis A., Charistos D., Anousis I. (1995). Sci. Total Environ..

[cit58] Zhao W., Lin X., Cai H., Mu T., Luo X. (2017). Ind. Eng. Chem. Res..

[cit59] Zhang Y., Yue X., Xu W., Zhang H., Li F. (2019). J. Hazard. Mater..

[cit60] Foo K. Y., Hameed B. H. (2010). Chem. Eng. J..

[cit61] Allen S. J., Mckay G., Porter J. F. (2004). J. Colloid Interface Sci..

[cit62] Weber T. W., Chakravorti R. K. (1974). AIChE J..

[cit63] Kusrini E., Wicaksono W., Gunawan C., Daud N. Z. A., Usman A. (2018). J. Environ. Chem. Eng..

[cit64] Zhao X. R., Xu X., Jiang X. Y., Teng J., Yu J. G. (2019). Desalin. Water Treat..

[cit65] Bai R., Yang F., Zhang Y., Zhao Z., Liao Q., Chen P., Zhao P., Guo W., Cai C. (2018). Carbohydr. Polym..

[cit66] Tan L., Liu Q., Song D., Jing X., Liu J., Li R., Hu S., Liu L., Wang J. (2015). New J. Chem..

[cit67] Alam M. S., Gorman-Lewis D., Chen N., Safari S., Baek K., Konhauser K. O., Alessi D. S. (2018). Environ. Sci. Technol..

[cit68] Iftekhar S., Ramasamy D. L., Srivastava V., Asif M. B., Sillanpää M. (2018). Chemosphere.

[cit69] Zhang J., Wang Q. (2016). J. Cleaner Prod..

[cit70] Duckworth O. W., Martin S. T. (2001). Geochim. Cosmochim. Acta.

[cit71] Quilès F., Burneau A. (2000). Vib. Spectrosc..

[cit72] Barbosa H. F. G., Cavalheiro É. T. G. (2019). Int. J. Biol. Macromol..

[cit73] Tian G., Geng J., Jin Y., Wang C., Li S., Chen Z., Wang H., Zhao Y., Li S. (2011). J. Hazard. Mater..

