# Supplementary material for: Mild hydrothermally treated brewer's spent grain for efficient removal of uranyl and rare earth metal ions
Source: RSC Adv. 2020 Dec 22;10(73):45116–29. doi: 10.1039/d0ra08164g (PMC9058606; doi:10.1039/d0ra08164g)
Supplement: RA-010-D0RA08164G-s001 [file RA-010-D0RA08164G-s001.pdf]

Electronic Supplementary Material (ESI) for RSC Advances.
This journal is © The Royal Society of Chemistry 2020

Supplementary information

Mild hydrothermal treated brewer's spent grain for efficient removal of uranyl and rare earth metal ions

Yi Su[a], Wendelin Böhm[b], Marco Wenzel[a], Silvia Paasch[c], Margret Acker[d], Thomas Doert,[e] Eike Brunner[c], Thomas Henle[b], Jan J. Weigand[a*]

[a] *Chair of Inorganic Molecular Chemistry, TU Dresden, 01062 Dresden, Germany*

[b] *Chair of Food Chemistry, TU Dresden, 01062 Dresden, Germany*

[c] *Chair of Bioanalytical Chemistry, TU Dresden, 01062 Dresden, Germany*

[d] *Central Radionuclide Laboratory, TU Dresden, 01062 Dresden, Germany*

[e] *Chair of Inorganic Chemistry II, TU Dresden, 01062 Dresden, Germany.*

Table list

Table S1 Parameters of the mashing process to produce BSG.
Table S2 Elemental analysis of BSG-based adsorbents.
Table S3 Mineral elements contents of BSG-based adsorbents.
Table S4 STA-GC-MS results of BSG (375−385 °C), ABSG (375−385 °C) and Yb-ABSG (375−385 °C). For STA, 20 °C/min, helium atmosphere. For GC, 35 °C for 3 min, increased with 5 °C/min until 220 °C, and hold at 220 °C for 3 min.

Figure list

Fig. S1 Raw FT-IR spectra of BSG-based adsorbents with varied (a) reaction temperature, (b) reaction time and with (c) metal adsorption. For ion-loading: 50 mg ABSG-150 °C, 16 h/ 50 mL solution, pH(La^{3+}, Eu^{3+}, Yb^{3+}) = 5.7, pH(UO_2^{2+}) = 4.7, c_0(La^{3+}, Eu^{3+}, Yb^{3+}) = 100 mg/L, c_0(UO_2^{2+}) = 300 mg/L, t = 1 h, room temperature.
Fig. S2 Raw FT-IR spectra of BSG and ABSG-150 °C, 16 h with bands position.
Fig. S3 Chemical structure of investigated MRPs.
Fig. S4 Pictures of BSG (left) and ABSG-150 °C, 16 h (right).
Fig. S5 Adsorption capacity of La^{3+}, Eu^{3+}, Yb^{3+} and UO_2^{2+} onto ABSG obtained employing air (ABSG-Air) and argon (ABSG-Ar) atmosphere. For hydrothermal treatment: 150 °C, 16 h. For adsorption: 2 mg adsorbent/ 2 mL solution, c_0(La^{3+}, Eu^{3+}, Yb^{3+}) = 100 mg/L, c_0(UO_2^{2+}) = 300 mg/L, pH(La^{3+}, Eu^{3+}, Yb^{3+}) = 5.7, pH(UO_2^{2+}) = 4.7, 1 h, room temperature.
Fig. S6 Comparison of Yb^{3+} adsorption capacity onto BSG and ABSG determined by ICP method and radiotracer method (1mg adsorbent/ 1 mL solution, c_0(Yb^{3+}) = 100 mg/L, pH= 5.5, 2 h, room temperature).
Fig. S7 Effect of the starting pH on the difference pH of BSG and ABSG to determine the point of zero charge (pH_{PZC}).
Fig. S8 Comparison of different adsorbents in present study and literature.[1–17]

*Corresponding author
E-mail address: jan.weigand@tu-dresden.de (J.J. Weigand)

Fig. S9 Adsorption isotherms of (a) La^{3+} onto BSG, (b) La^{3+} onto ABSG, (c) Eu^{3+} onto BSG, (d) Eu^{3+} onto ABSG, (e) Yb^{3+} onto BSG, (f) Yb^{3+} onto ABSG, (g) UO_2^{2+} onto BSG and (h) UO_2^{2+} onto ABSG. For adsorption, 2 mg adsorbent/ 2 mL metal solution, c_0 = 100−600 mg/L, t_{BSG} = 2 h, t_{ABSG} = 1 h, pH(UO_2^{2+}) = 4.7, pH(La^{3+}, Eu^{3+}, Yb^{3+}) = 5.7, T = 25 ℃, 45 ℃, 65 ℃, stirrer speed = 180 rpm.
Fig. S10 Langmuir model fitting of isotherms of (a) La^{3+} onto BSG, (b) La^{3+} onto ABSG, (c) Eu^{3+} onto BSG, (d) Eu^{3+} onto ABSG, (e) Yb^{3+} onto BSG, (f) Yb^{3+} onto ABSG, (g) UO_2^{2+} onto BSG and (h) UO_2^{2+} onto ABSG.
Fig. S11 $\ln K_e^0$ versus 1/T plot for thermodynamic parameters calculation (a) BSG and (b) ABSG.
Fig. S12 (a-c) SEM image (1000x), EDX element mapping (20 kV/10 µA, 5000x, 25 frames) and distribution of La on La loaded-ABSG, (d-f) SEM image (1000x), EDX mapping (20 kV/10 µA, 1000x, 25 frames) and distribution of Eu on Eu loaded-ABSG and (g-i) SEM image (1000x), EDX mapping (20 kV/10 µA, 5000x, 25 frames) and distribution of Yb on Yb loaded-ABSG. For ion-loading: 50 mg ABSG/ 50 mL solution, pH= 5.7, c_0= 100 mg/L, t = 1 h, room temperature.

Table S1 Parameters of the mashing process to produce BSG.

Procedure	Initial temperature (℃)	End temperature (℃)	Time (min)
Mash-in	55	54.8	10
Protein rest	62	61.8	30
Maltose rest	68	67.8	10
Sugar rest	72	71.8	25
Mash-out	78	77.8	10

Table S2 Elemental analysis of BSG-based adsorbents.

Sample	N (%)	C(%)	H(%)	S(%)	Mineral(%)	O(%)
BSG	5.1	49.1	6.1	0.3	1.3	38.1
ABSG-1 h, 150 ℃	4.9	47.4	6.5	0.4	1.3	39.4
ABSG-4 h, 150 ℃	4.5	48.5	7.6	0.2	1.3	38.0
ABSG-8 h, 150 ℃	4.2	49.4	7.8	0.0	1.3	37.3
ABSG-16 h, 150 ℃	5.0	53.4	6.8	0.3	1.6	32.8
ABSG-24 h, 150 ℃	4.6	60.8	6.8	0.4	0.4	27.0
ABSG-16 h, 100 ℃	4.4	48.2	7.3	0.2	1.2	38.7
ABSG-16 h, 125 ℃	4.2	48.2	7.1	0.3	1.3	38.9
ABSG-16 h, 175 ℃	4.1	63.0	6.2	0.3	0.3	26.0

Table S3 Mineral elements contents of BSG-based adsorbents.

Sample	Ca (mg/kg)	Mg (mg/kg)	P (mg/kg)	Si (mg/kg)	Fe (mg/kg)	K (mg/kg)	Mn (mg/kg)	Na (mg/kg)	Zn (mg/kg)
BSG	2669 ± 7	2509 ± 27	5861 ± 103	791 ± 7	240 ± 4	849 ± 9	41 ± 1	166 ± 11	139 ± 4
ABSG 150 ℃, 1 h	2321 ± 43	2627 ± 48	5895 ± 56	625 ± 15	155 ± 1	816 ± 9	36 ± 0.1	92 ± 5	98 ± 4
ABSG 150 ℃, 4 h	2481 ± 10	2714 ± 7	6066 ± 20	1033 ± 9	177 ± 3	729 ± 3	39 ± 1	58 ± 6	96 ± 2
ABSG 150 ℃, 8 h	3065 ± 86	2343 ± 54	5127 ± 134	974 ± 36	195 ± 7	664 ± 18	39 ± 0.4	67 ± 5	122 ± 1
ABSG 150℃, 16 h	2717 ± 4	3201 ± 30	7737 ± 2	1077 ±12	293 ± 0.1	1083 ± 12	52 ± 0.1	137 ± 2	154 ± 1
ABSG 150 ℃,24 h	2549 ± 62	191 ± 15	532 ± 44	281 ± 48	219 ± 12	120 ± 5	165 ± 3	62 ± 4	94 ± 1
ABSG 100 ℃, 16 h	2174 ± 37	2205 ± 5	5283 ± 25	894 ± 14	163 ± 3	683 ± 8	35 ± 0.1	65 ± 2	105 ± 1
ABSG 125 ℃, 16 h	2545 ± 42	2630 ± 51	5838 ± 107	1081 ± 12	159 ± 4	773 ± 14	41 ± 1	68 ± 5	124 ± 1
ABSG 175 ℃, 16 h	1228 ± 35	333 ± 10	787 ± 68	288 ± 17	322 ± 7	53 ± 2	21 ± 0.2	170 ± 6	166 ± 2

Errors are those obtained from the ICP measurements.

Table S4 STA-GC-MS results of BSG (375−385 ℃), ABSG (375−385 ℃) and Yb-ABSG (340−360 ℃). For STA, 20 ℃/min, helium atmosphere. For GC, 35 °C for 3 min, increased with 5 ℃/min until 220 ℃, and hold at 220 ℃ for 3 min.

Name	Retention time (GC, min)	Formula	BSG, Area %	ABSG, Area %	Yb-ABSG, Area %
Carbondioxide	9.51	CO_2	67.7	48.6	41.2
Water	9.78	H_2O	25.4	43.8	39.7
Acetaldehyde	10.14	C_2H_4O	0.2	0.2	0.3
Acetic anhydride	11.25	$C_4H_6O_5$	0.7	0.5	2.7
Hydroxyacetaldehyde	12.43	$C_2H_4O_2$	0.3	0.1	4.9
Acetic acid	13.04	$C_2H_4O_2$	0	1.3	2.7
2-Butanone	13.49	C_4H_8O	0.4	0.3	0.8
3-Methylfuran	13.93	C_5H_6O	1.5	1.8	1.3
2-methylfuran	14.01	C_5H_6O	0.3	0.4	0.3
1-Hydroxypropan-2-one	15.14	$C_3H_6O_2$	1.2	0.2	1.9
3-Penten-2-one	15.92	C_5H_8O	0.5	0.6	0.6
2,5-Dimethylfuran	17.38	C_6H_8O	0.9	1.0	0.9
Pyridin	18.62	C_5H_5N	0	0.6	1.1
Furfural	21.79	$C_5H_4O_2$	0.2	0.2	0.6
1,2-Cyclopentanedione	25.48	$C_5H_6O_2$	0.7	0.5	0.9

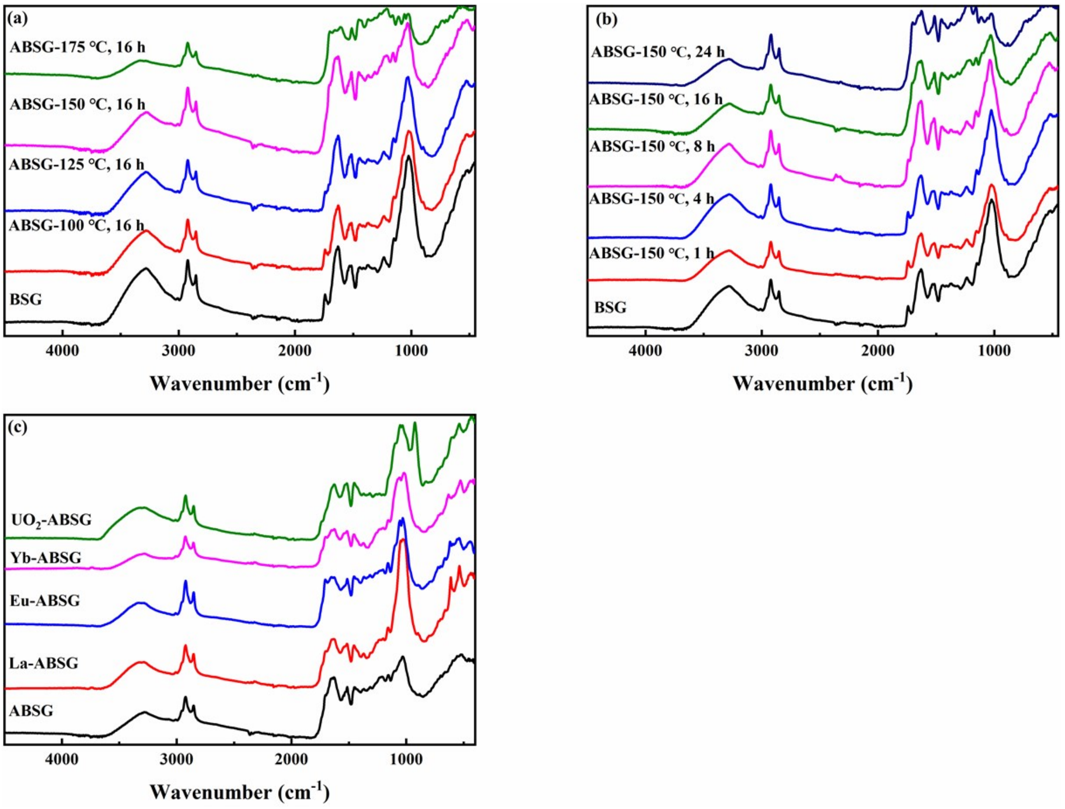

Fig. S1 Raw FT-IR spectra of BSG-based adsorbents with varied (a) reaction temperature, (b) reaction time and with (c) metal adsorption. For ion-loading: 50 mg ABSG-150 °C, 16 h/ 50 mL solution, pH(La^{3+}, Eu^{3+}, Yb^{3+}) = 5.7, pH(UO_2^{2+}) = 4.7, c_0(La^{3+}, Eu^{3+}, Yb^{3+}) = 100 mg/L, c_0(UO_2^{2+}) = 300 mg/L, t = 1 h, room temperature.

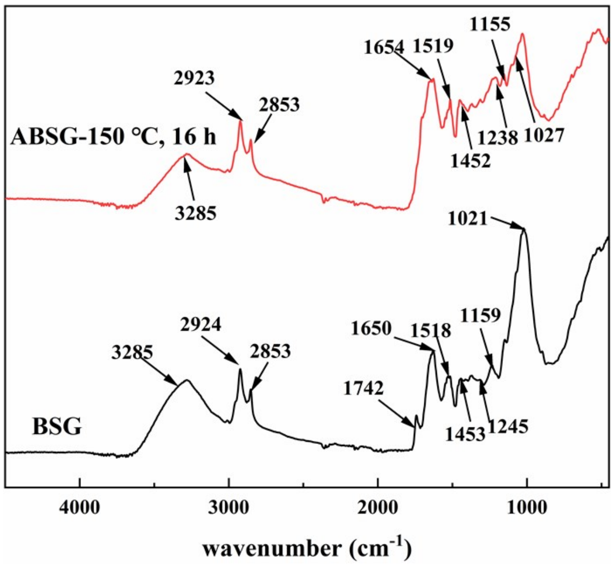

Fig. S2 Raw FT-IR spectra of BSG and ABSG-150 ℃, 16 h with bands position.

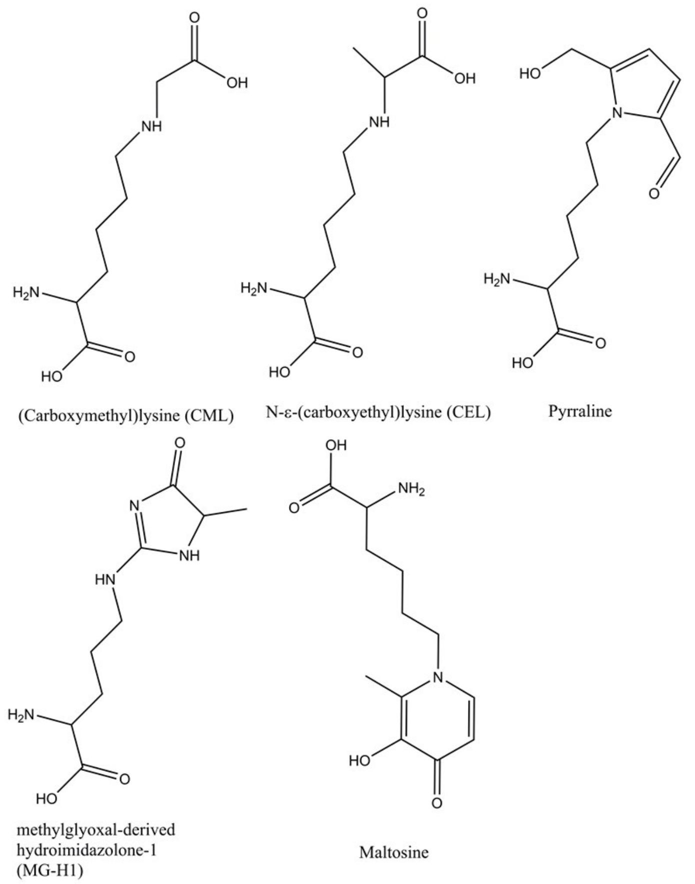

Fig. S3 Chemical structure of investigated MRPs.

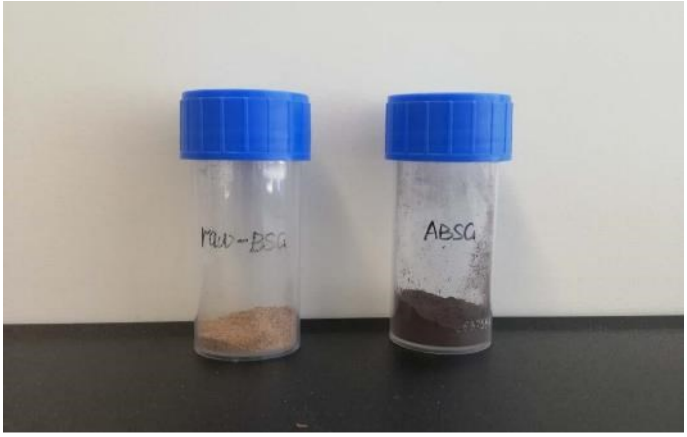

Fig. S4 Pictures of BSG (left) and ABSG-150 ℃, 16 h (right).

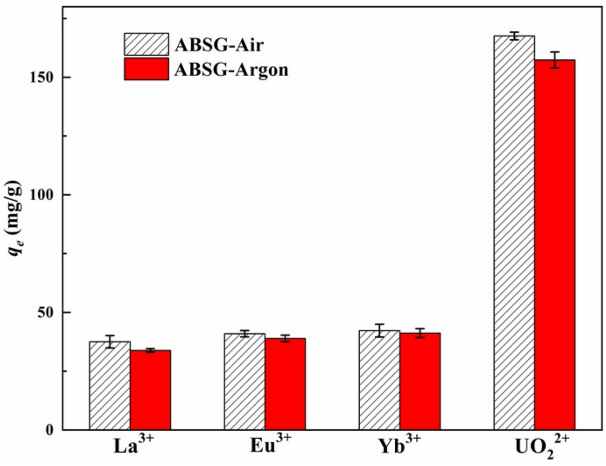

Fig. S5 Adsorption capacity of La^{3+}, Eu^{3+}, Yb^{3+} and UO_2^{2+} onto ABSG obtained employing air (ABSG-Air) and argon (ABSG-Ar) atmosphere. For hydrothermal treatment: 150 ℃, 16 h. For adsorption: 2 mg adsorbent/ 2 mL solution, $c_0(La^{3+}, Eu^{3+}, Yb^{3+})$ = 100 mg/L, $c_0(UO_2^{2+})$ = 300 mg/L, pH(La^{3+}, Eu^{3+}, Yb^{3+}) = 5.7, pH(UO_2^{2+}) = 4.7, 1 h, room temperature.

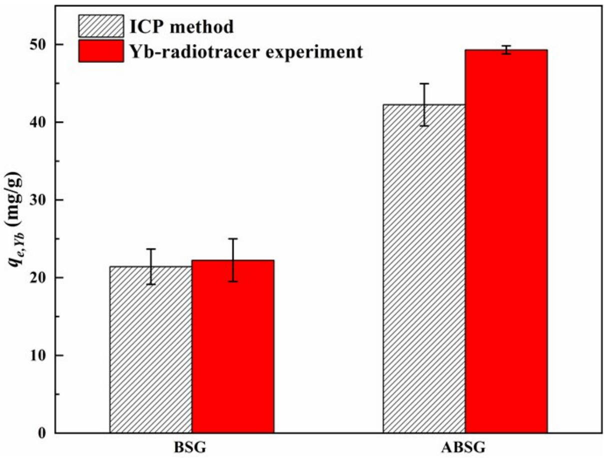

Fig. S6 Comparison of Yb^{3+} adsorption capacity onto BSG and ABSG determined by ICP method and radiotracer method (1mg adsorbent/ 1 mL solution, $c_0(Yb^{3+})$ = 100 mg/L, pH= 5.5, 2 h, room temperature).

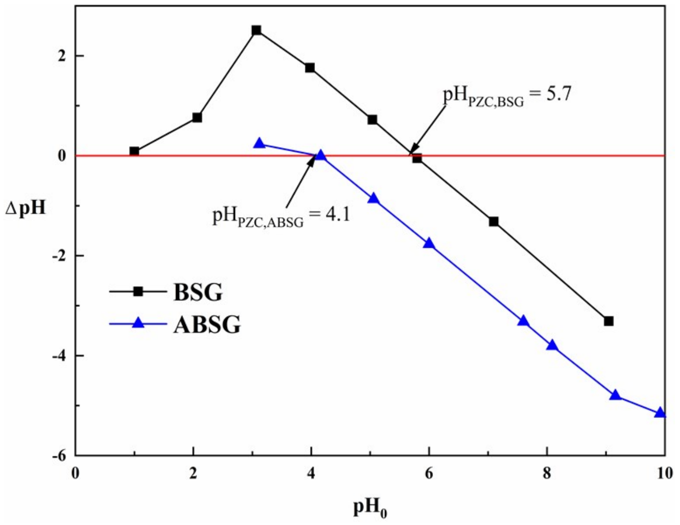

Fig. S7 Effect of the starting pH on the difference pH of BSG and ABSG to determine the point of zero charge (pH_{PZC}).

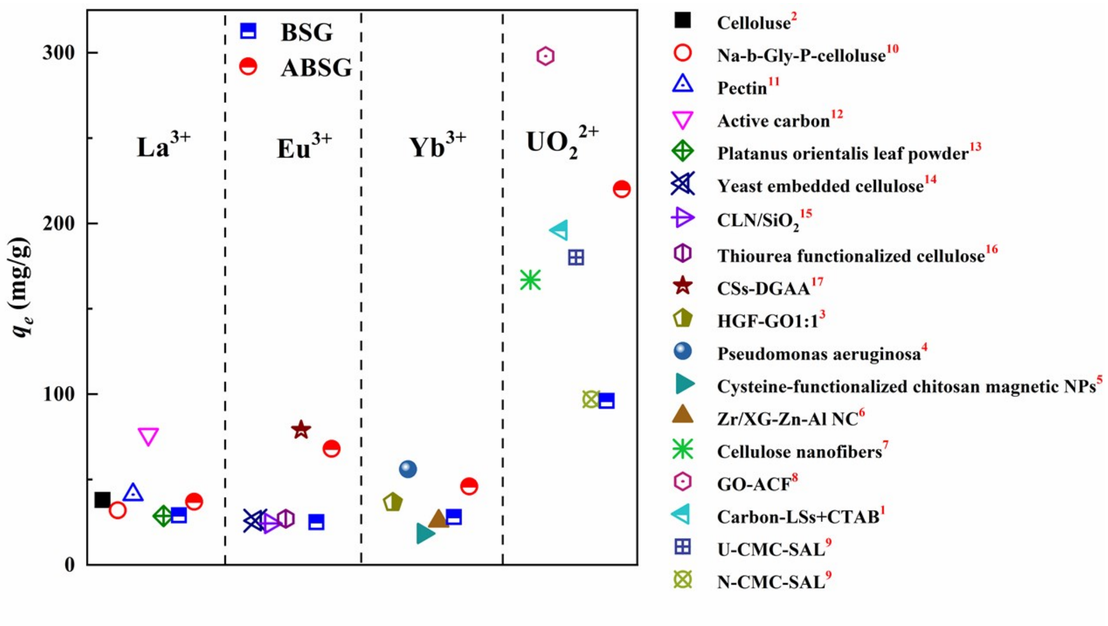

Fig. S8 Comparison of different adsorbents in present study and literature.[1–17]

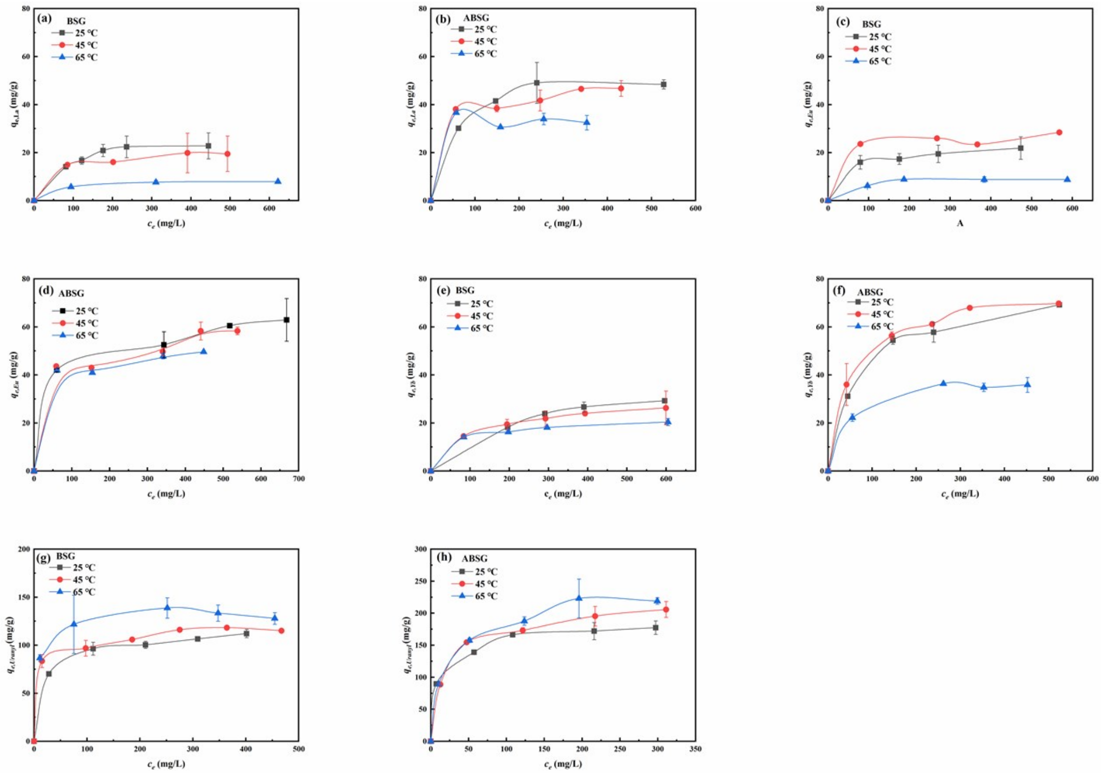

Fig. S9 Adsorption isotherms of (a) La^{3+} onto BSG, (b) La^{3+} onto ABSG, (c) Eu^{3+} onto BSG, (d) Eu^{3+} onto ABSG, (e) Yb^{3+} onto BSG, (f) Yb^{3+} onto ABSG, (g) UO_2^{2+} onto BSG and (h) UO_2^{2+} onto ABSG. For adsorption, 2 mg adsorbent/ 2 mL metal solution, c_0 = 100−600 mg/L, t_{BSG} = 2 h, t_{ABSG} = 1 h, pH(UO_2^{2+}) = 4.7, pH(La^{3+}, Eu^{3+}, Yb^{3+}) = 5.7, T = 25 ℃, 45 ℃, 65 ℃, stirrer speed = 180 rpm.

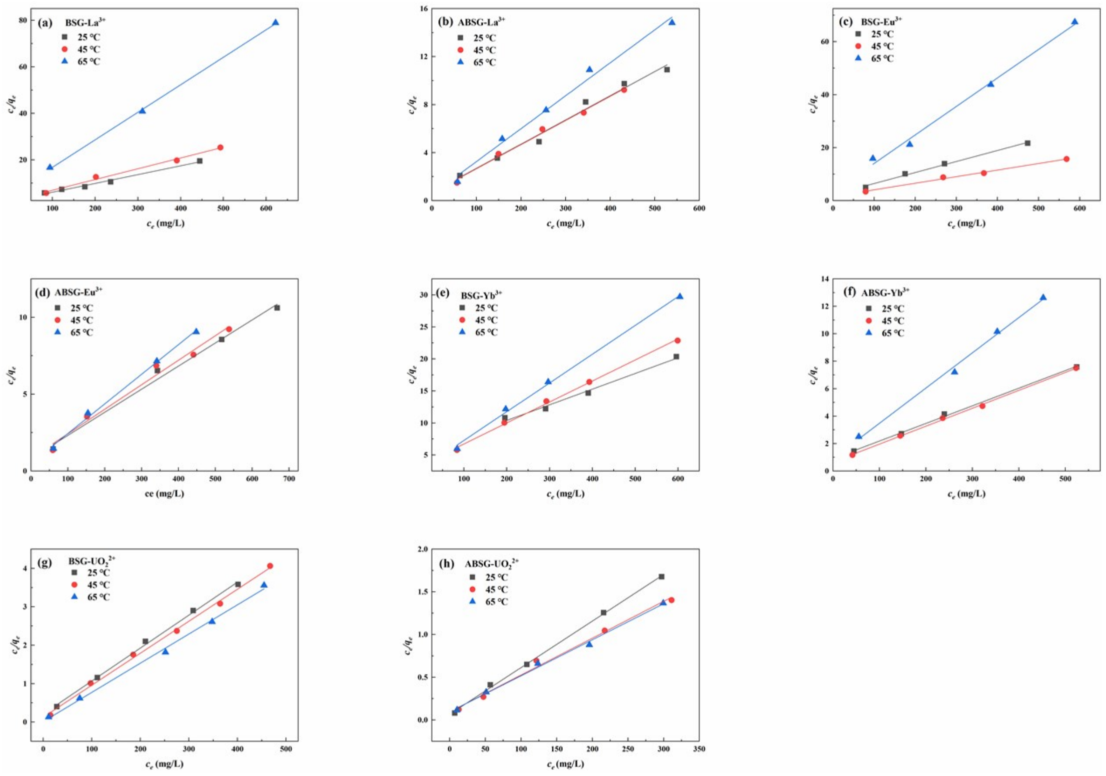

Fig. S10 Langmuir model fitting of isotherms of (a) La^{3+} onto BSG, (b) La^{3+} onto ABSG, (c) Eu^{3+} onto BSG, (d) Eu^{3+} onto ABSG, (e) Yb^{3+} onto BSG, (f) Yb^{3+} onto ABSG, (g) UO_2^{2+} onto BSG and (h) UO_2^{2+} onto ABSG.

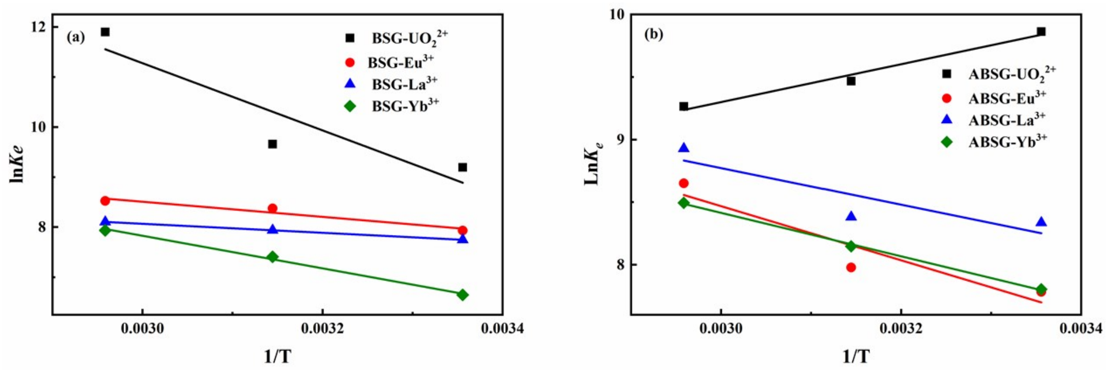

Fig. S11 lnK_e^0 versus 1/T plot for thermodynamic parameter calculations for (a) BSG and (b) ABSG.

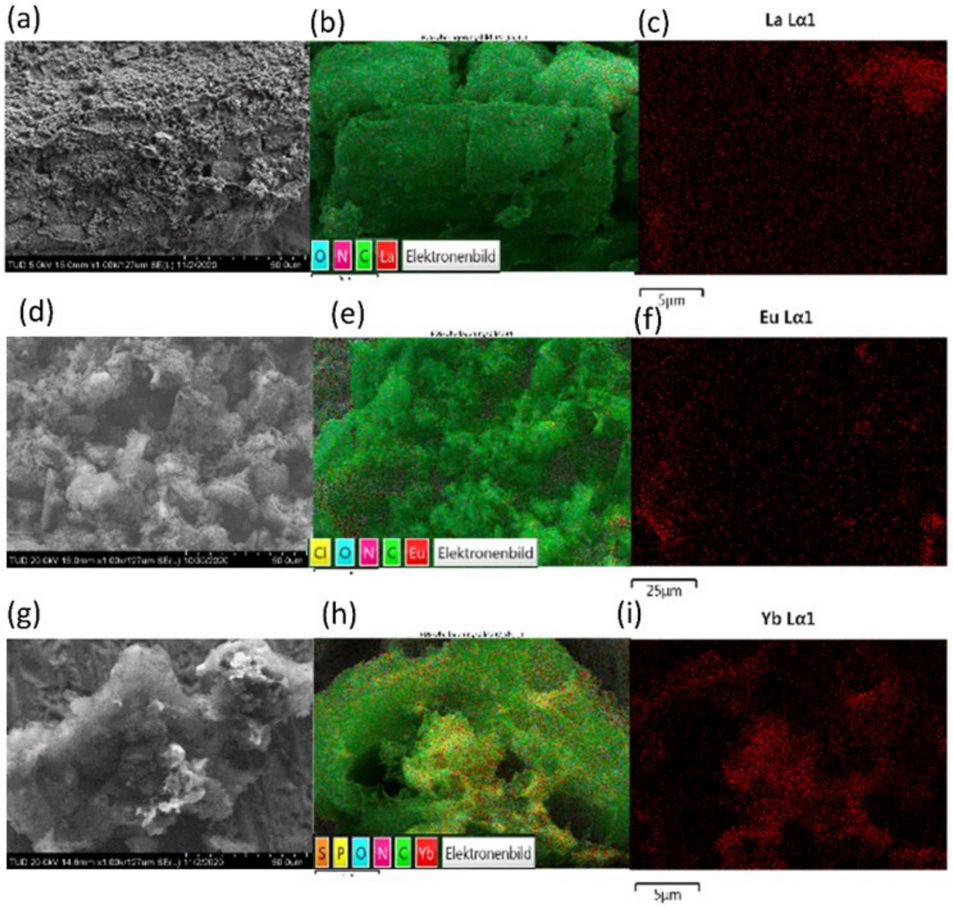

Fig. S12 (a-c) SEM image (1000x), EDX element mapping (20 kV/10 μA, 5000x, 25 frames) and distribution of La on La loaded-ABSG, (d-f) SEM image (1000x), EDX mapping (20 kV/10 μA, 1000x, 25 frames) and distribution of Eu on Eu loaded-ABSG and (g-i) SEM image (1000x), EDX mapping (20 kV/10 μA, 5000x, 25 frames) and distribution of Yb on Yb loaded-ABSG. For ion-loading: 50 mg ABSG/ 50 mL solution, pH= 5.7, c_0= 100 mg/L, t = 1 h, room temperature.

Reference

1 W. Zhao, X. Lin, H. Cai, T. Mu and X. Luo, *Ind. Eng. Chem. Res.*, 2017, **56**, 12745–12754.

2 A. A. Tolba, S. I. Mohamady, S. S. Hussin, T. Akashi, Y. Sakai, A. A. Galhoum and E. Guibal, *Carbohydr. Polym.*, 2017, **157**, 1809–1820.

3 X. Xu, J. Zou, J. Teng, Q. Liu, X. Y. Jiang, F. P. Jiao, J. G. Yu and X. Q. Chen, *Ecotoxicol. Environ. Saf.*, 2018, **166**, 1–10.

4 A. C. Texier, Y. Andrès and P. Le Cloirec, *Environ. Sci. Technol.*, 1999, **33**, 489–495.

5 A. A. Galhoum, M. G. Mafhouz, S. T. Abdel-Rehem, N. A. Gomaa, A. A. Atia, T. Vincent and E. Guibal, *Nanomaterials*, 2015, **5**, 154–179.

6 T. Kegl, A. Košak, A. Lobnik, Z. Novak, A. K. Kralj and I. Ban, *J. Hazard. Mater.*, 2020, **386**, 121632.

7 H. Ma, B. S. Hsiao and B. Chu, *ACS Macro Lett.*, 2012, **1**, 213–216.

8 S. Chen, J. Hong, H. Yang and J. Yang, *J. Environ. Radioact.*, 2013, **126**, 253–258.

9 M. Monier and D. A. Abdel-Latif, *Carbohydr. Polym.*, 2013, **97**, 743–752.

10 A. Gabor, C. M. Davidescu, A. Negrea, M. Ciopec, I. Grozav, P. Negrea and N. Duteanu, *J. Environ. Manage.*, 2017, **204**, 839–844.

11 E. Kusrini, W. Wicaksono, C. Gunawan, N. Z. A. Daud and A. Usman, *J. Environ. Chem. Eng.*, 2018, **6**, 6580–6588.

12 E. M. Iannicelli-Zubiani, P. Gallo Stampino, C. Cristiani and G. Dotelli, *Chem. Eng. J.*, 2018, **341**, 75–82.

13 Ş. Sert, C. Kütahyali, S. Inan, Z. Talip, B. Çetinkaya and M. Eral, *Hydrometallurgy*, 2008, **90**, 13–18.

14 B. Arunraj, T. Sathvika, V. Rajesh and N. Rajesh, *ACS Omega*, 2019, **4**, 940–952.

15 S. Iftekhar, V. Srivastava and M. Sillanpää, *Chem. Eng. J.*, 2017, **320**, 151–159.

16 A. Negrea, A. Gabor, C. M. Davidescu, M. Ciopec, P. Negrea, N. Duteanu and A. Barbulescu, *Sci. Rep.*, 2018, **8**, 1–11.

17 R. Bai, F. Yang, Y. Zhang, Z. Zhao, Q. Liao, P. Chen, P. Zhao, W. Guo and C. Cai, *Carbohydr. Polym.*, 2018, **190**, 255–261.
